# Supplementary material for: Identification of a common polymorphism in COQ8B acting as a modifier of thoracic aortic aneurysm severity
Source: HGG Adv. 2021 Sep 17;3(1):100057. doi: 10.1016/j.xhgg.2021.100057 (PMC8670066; doi:10.1016/j.xhgg.2021.100057)
Supplement: Document S1. Figures S1–S7 and Tables S1–S4 [file mmc1.pdf]

HGGA, Volume *3*

Supplemental information

Identification of a common polymorphism in *COQ8B* acting as a modifier of thoracic aortic aneurysm severity

Benjamin J. Landis, Dongbing Lai, Dong-Chuan Guo, Joel S. Corvera, Muhammad T. Idrees, Henry W. Stadler, Christian Cuevas, Gavin U. Needler, Courtney E. Vujakovich, Dianna M. Milewicz, Robert B. Hinton, and Stephanie M. Ware

Supplemental Figures.

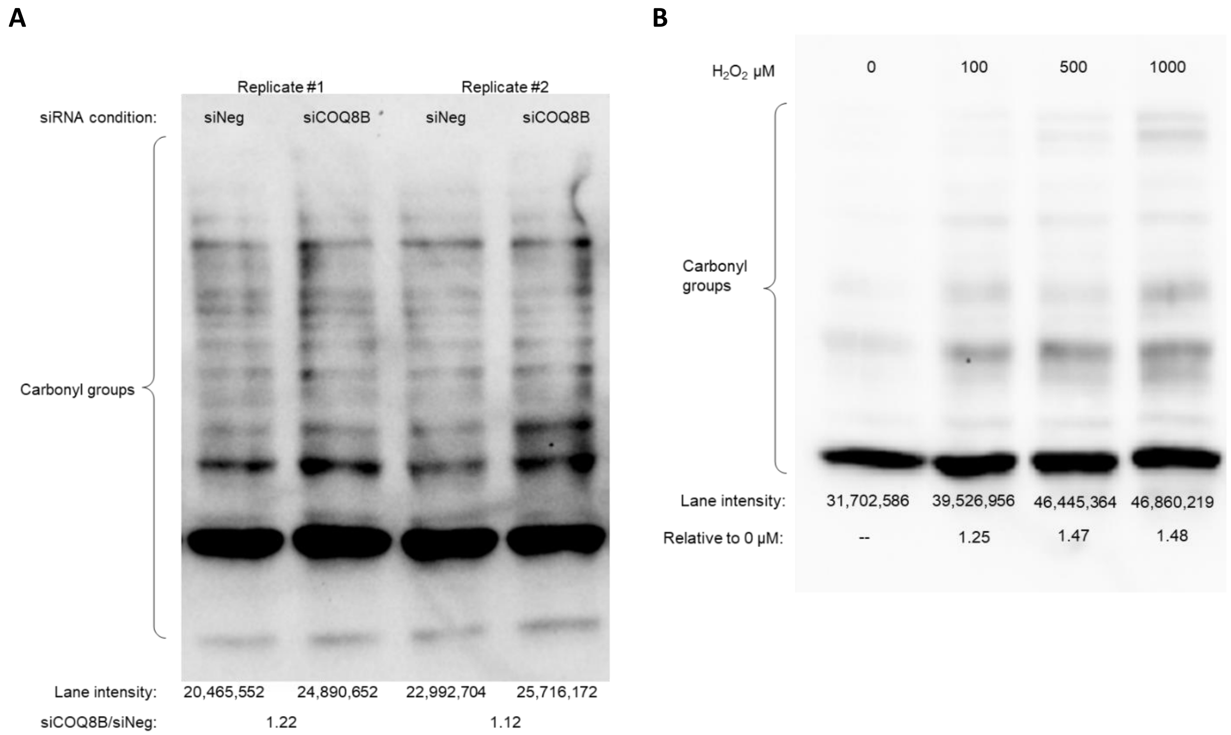

Figure S1. Replication of Oxyblot experiments and determination of assay sensitivity to oxidative stress.

(A) Protein carbonylation levels in healthy control aortic SMCs transfected with siRNA targeting COQ8B (siCOQ8B) versus negative control siRNA (siNeg). Chemiluminescent signal intensity is shown below each lane (Lane intensity). These represent biological replicates of the Oxyblot result for Control #1 SMCs that is shown in Figure 1E.

(B) Protein carbonylation levels in whole cell lysate protein samples from healthy control aortic SMCs administered different dosages of H_2O_2 (0, 100, 500, or 1000 μM) for 24 hour duration.

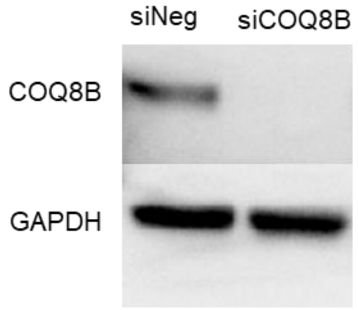

Figure S2. Western blots in whole cell lysates using a second COQ8B antibody.
Western blot for COQ8B using Atlas antibody in whole cell lysate proteins from SMCs treated with negative control siRNA versus siCOQ8B. Result confirms the specificity of LSBio antibody for COQ8B.

A

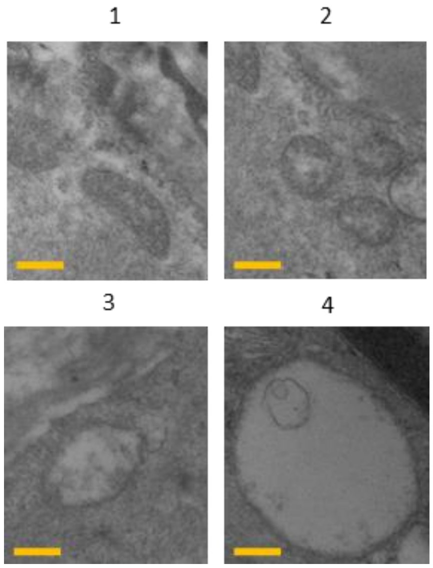

B

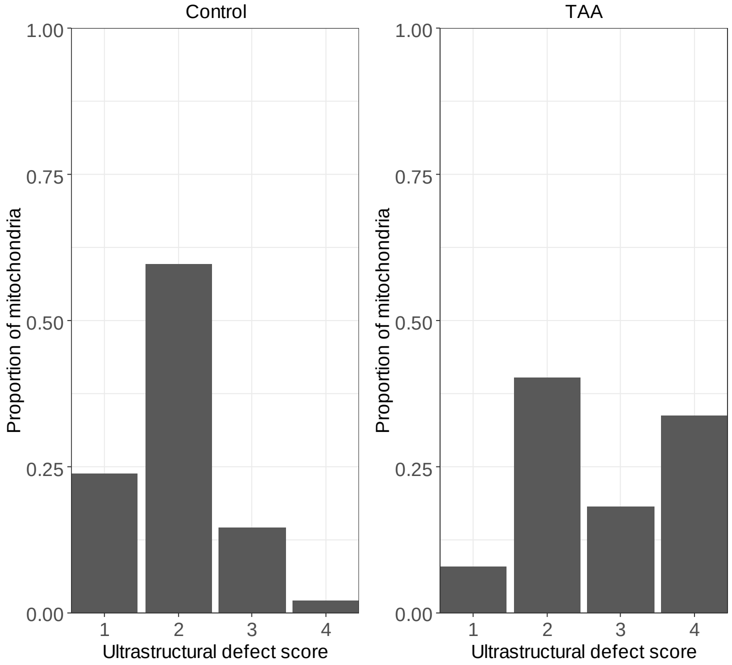

Figure S3. Scoring of ultrastructural defects of mitochondria using transmission electron microscopy of aortic tissues.

(A) Examples of mitochondria with ultrastructural defect scores of 1 (intact cristae), 2 (irregular cristae), 3 (deficient cristae), and 4 (swollen and deficient cristae).

(B) Distributions of ultrastructural defect scores in control and TAA mitochondria. Yellow bar in panel A = 250 nm.

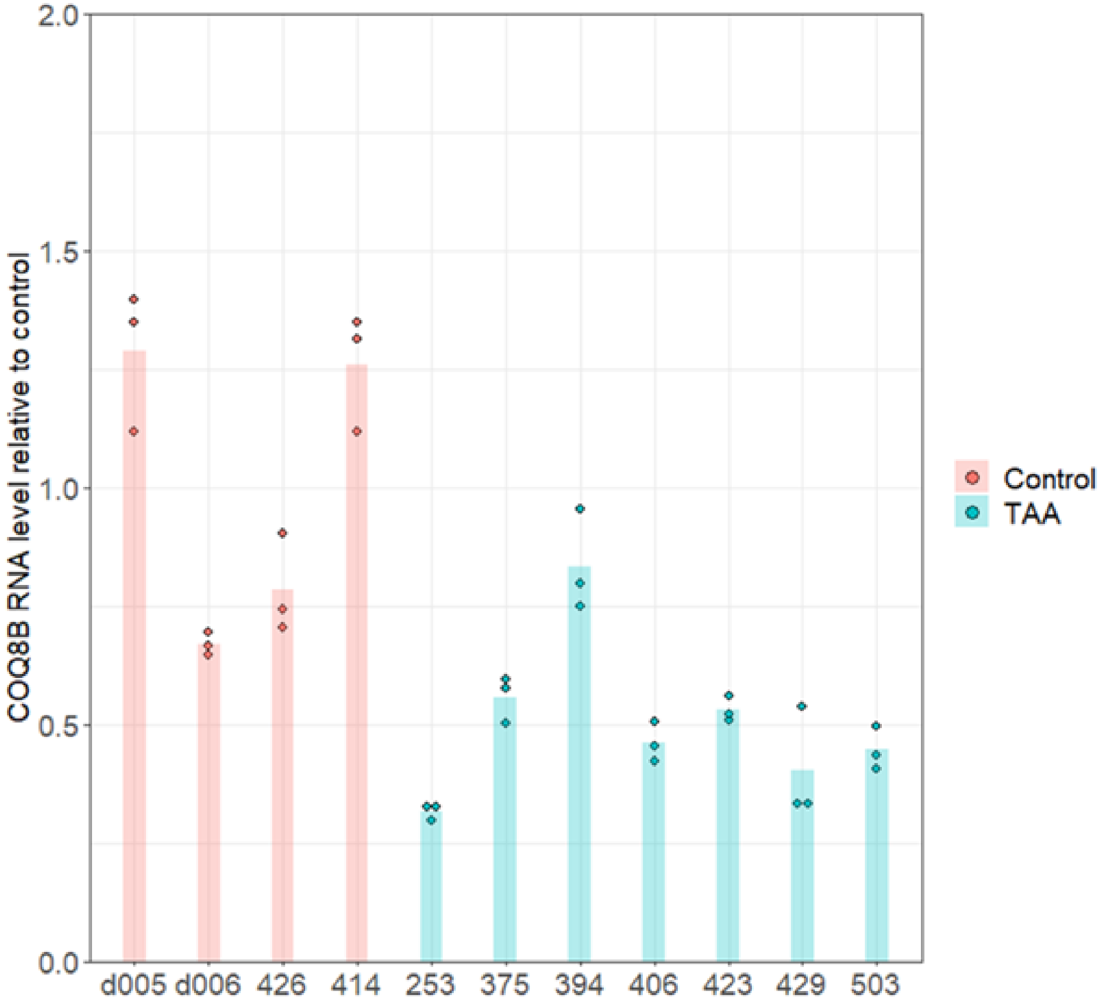

Figure S4. RNA levels of *COQ8B* between TAA (N=7 cases) and controls (N=4 individuals) in cultured proximal aortic SMCs. *COQ8B* RNA levels were adjusted for *ACTB* and are displayed relative to mean of control SMCs. Each point corresponds to an independent well of SMCs and bars indicate mean for each individual. These mean values are shown in Figure 3F.

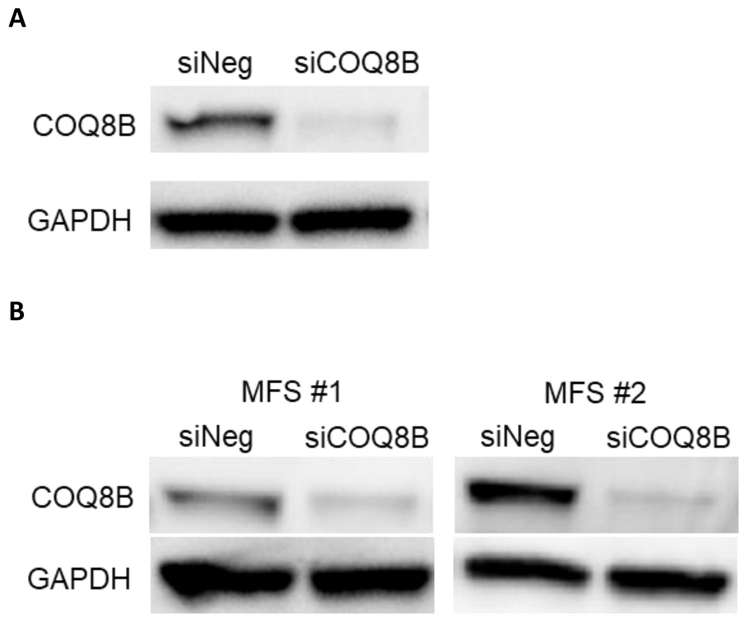

Figure S5. Western blots for COQ8B in knockdown experiments.
(A) Western blot for COQ8B in the mitochondrial protein fractions of SMCs from a participant with MFS transfected with siNeg versus siCOQ8B.
(B) Western blot for COQ8B and GAPDH in the siNeg and siCOQ8 whole cell protein samples used for Oxyblot in Figure 4A.

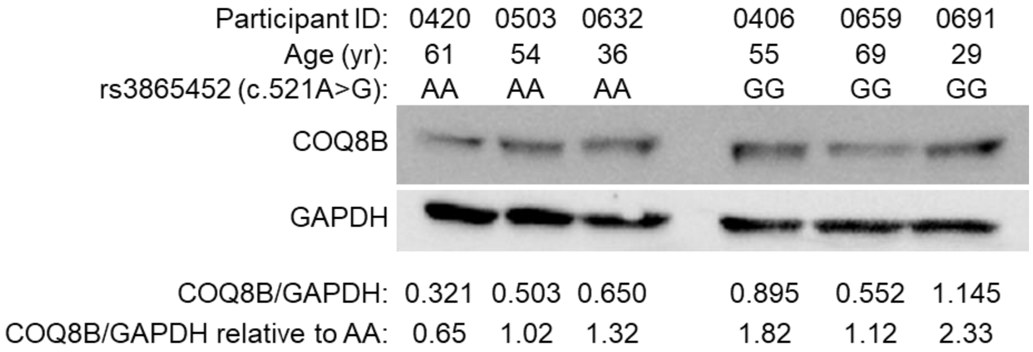

Figure S6. Western blot of aortic SMC protein lysates for 6 additional participants included in Figure 7A.

The ratios of chemiluminescent signal intensity between COQ8B and GAPDH bands are shown for each sample and also shown relative to the mean for AA samples.

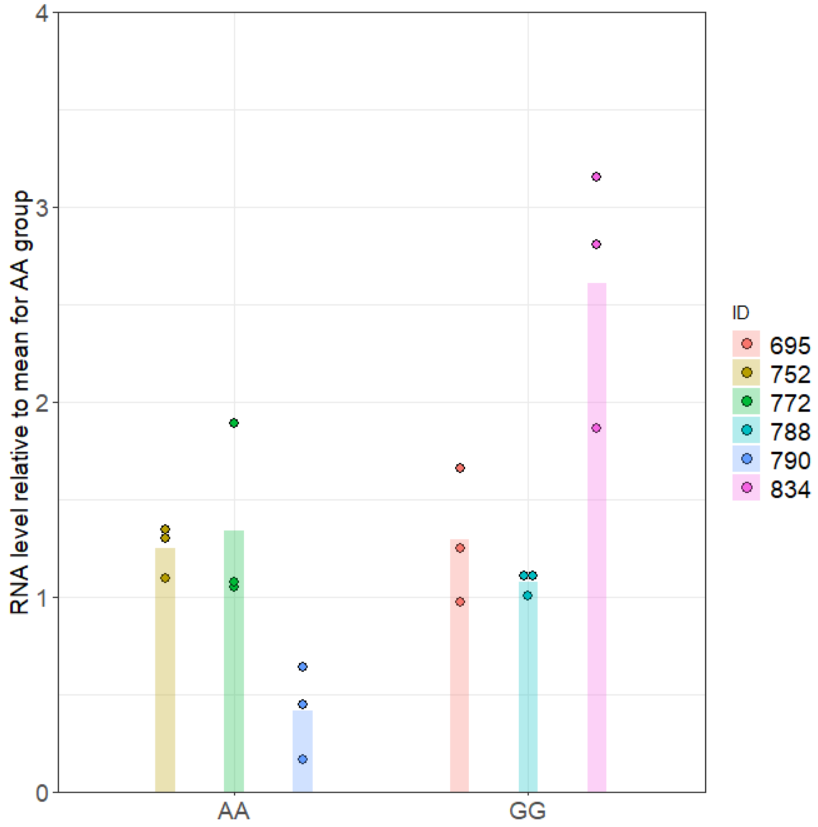

Figure S7. *COQ8B* RNA levels in TAA SMCs from participants homozygous for rs3865452 (GG) or the reference allele (AA).

RNA extractions were performed at 2nd passage in parallel with protein extractions that were used for Western blot in Figure 5. No significant difference in *COQ8B* RNA levels was observed between AA and GG groups.

Supplemental Tables.

Table S1. Primers used in qRT-PCR analysis.

Gene	Primer sequences
COQ8B	GTGTGACTACCGTCGTGAGG<br>GGAAGAAGGGGTCATTTGC
ACTA2	CTGTTCCAGCCATCCTTCAT<br>TCATGATGCTGTTGTAGGTGGT
CNN1	GCTGTCAGCCGAGGTTAAGA<br>CCCTCGATCCACTCTCTCAG
MYOCD	CAAGTTCAGCAATTTCAGAGGTAA<br>TGACTCCGGGTCATTTGC

Table S2. Clinical and demographic data of participants whose ascending aortic tissues were studied with transmission electron microscopy.

Characteristic	Control (N=3)	TAA (N=7)
Age (years), mean ± standard deviation	36 ± 25	58 ± 10
Sex, N		
Male	3	6
Female	0	1
Race, N		
White	2	6
Black	0	1
Data not available	1	0
Family history of TAA, N	0	3
Thoracic aortic dissection, N	0	1
Aortic valve morphology, N		
Tricuspid	3	5
Bicuspid or unicuspid	0	2
Hypertension, N	0	3
Dyslipidemia, N	1	2
Type 2 diabetes, N	1	0
Coronary artery disease, N	1	1

TAA: thoracic aortic aneurysm

Table S3. Clinical and demographic data of participants whose samples were used to compare *COQ8B* RNA levels in primary culture aortic SMCs between TAA and controls.

Characteristic	Control (N=4)	TAA (N=7)
Age, years (mean ± standard deviation)	26 ± 10	61 ± 13
Sex, N		
Male	4	3
Female	0	4
Race, N		
White	2	6
Black	0	1
Data not available	2	0
Family history of TAA, N	0	1
Thoracic aortic dissection, N	0	0
Aortic valve morphology, N		
Tricuspid	3	4
Bicuspid or unicuspid	1	3
Hypertension, N	1	7
Dyslipidemia, N	0	3

TAA: thoracic aortic aneurysm.

Table S4. Cardiovascular characteristics of patients in longitudinal TAA cohort used for mixed model analysis.

Characteristic	MFS or LDS (N=23)	Nonsyndromic (N=25)
Initial aortic root Z-score, mean ± standard deviation	3.8 ± 1.8	3.5 ± 1.7
Initial ascending aorta Z-score, mean ± standard deviation	0.5 ± 1.4	2.8 ± 2.0
Age at initial echo (years), mean ± standard deviation	10.1 ± 5.9	9.9 ± 4.1
Duration of follow up (years), mean ± standard deviation	5.6 ± 4.9	5.1 ± 3.2
Number of echocardiograms, mean ± standard deviation	7.4 ± 4.6	6.4 ± 3.4
Bicuspid aortic valve, N	3	11
Aortic replacement surgery, N	2	1
Aortic dissection, N	1	0
