# Supplementary material for: Identification of a common polymorphism in COQ8B acting as a modifier of thoracic aortic aneurysm severity
Source: HGG Adv. 2021 Sep 17;3(1):100057. doi: 10.1016/j.xhgg.2021.100057 (PMC8670066; doi:10.1016/j.xhgg.2021.100057)
Supplement: Document S2. Article plus supplemental information [file mmc2.pdf]

HGG Advances

ARTICLE

Identification of a common polymorphism in *COQ8B* acting as a modifier of thoracic aortic aneurysm severity

Benjamin J. Landis,[1,2,*] Dongbing Lai,[2] Dong-Chuan Guo,[3] Joel S. Corvera,[4] Muhammad T. Idrees,[5] Henry W. Stadler,[1] Christian Cuevas,[1] Gavin U. Needler,[1] Courtney E. Vujakovich,[1] Dianna M. Milewicz,[3] Robert B. Hinton,[6] and Stephanie M. Ware[1,2]

Summary

Thoracic aortic aneurysm (TAA) predisposes to sudden, life-threatening aortic dissection. The factors that regulate interindividual variability in TAA severity are not well understood. Identifying a molecular basis for this variability has the potential to improve clinical risk stratification and advance mechanistic insight. We previously identified *COQ8B*, a gene important for biosynthesis of coenzyme Q, as a candidate genetic modifier of TAA severity. Here, we investigated the physiological role of *COQ8B* in human aortic smooth muscle cells (SMCs) and further tested its genetic association with TAA severity. We find COQ8B protein localizes to mitochondria in SMCs, and loss of mitochondrial COQ8B leads to increased oxidative stress, decreased mitochondrial respiration, and altered expression of SMC contractile genes. Oxidative stress and mitochondrial cristae defects were prevalent in the medial layer of human proximal aortic tissues in individuals with TAA, and *COQ8B* expression was decreased in TAA SMCs compared with controls. A common single nucleotide polymorphism (SNP) rs3865452 in *COQ8B* (c.521A>G [p.His174Arg]) was associated with decreased rate of aortic root dilation in young individuals with TAA. In addition, the SNP was less frequent in a second cohort of early-onset thoracic aortic dissection (TAD) cases compared with controls. COQ8B protein levels in aortic SMCs were increased in TAA individuals homozygous for rs3865452 compared with those homozygous for the reference allele. Thus, *COQ8B* is important for aortic SMC metabolism, which is dysregulated in TAA, and rs3865452 may decrease TAA severity by increasing the COQ8B level. Genotyping rs3865452 may be useful for clinical risk stratification and tailored aortopathy management.

Introduction

Thoracic aortic aneurysm (TAA) is characterized by degeneration of the aortic medial layer and predisposes to life-threatening thoracic aortic dissection (TAD). Recent epidemiological data indicate that TAA causes approximately 12,000 deaths annually in the United States.[1] The genetic causes of TAA include Mendelian dominant connective tissue disorders such as Marfan syndrome (MFS [MIM: 154700]) and Loeys-Dietz syndrome (LDS) (LDS1/2 [MIM: 609192/610168]), as well as heritable nonsyndromic etiologies. Bicuspid aortic valve (BAV), which has a heritable basis, is also associated with TAA development and TAD.

Disease progression in TAA is occult, and onset of TAD is sudden. Therefore, precise stratification of risk is necessary to optimize clinical decision making for individuals with TAA or associated genetic diagnoses. Specific recommendations for TAA management exist for genetic diagnoses such as MFS or LDS and provide the rationale for using genetics to risk stratify individuals for progression or dissection.[2] However, the severity of TAA is highly variable, even within specific subgroups such as MFS or BAV.[3] TAA severity may significantly differ between family members who carry the same pathogenic variant.[4] Moreover, approximately half of individuals who have BAV ultimately do not develop TAA.[5] The factors that control the penetrance and interindividual variability in TAA development and severity are not understood. This gap in knowledge limits clinical risk stratification. We previously identified *COQ8B* (COQ8B [MIM: 615567]) (formerly named *ADCK4*) as a candidate genetic modifier of TAA severity in familial studies.[4] *COQ8B* is known to be important for the biosynthesis of coenzyme Q (CoQ), although the gene's precise biochemical role is not completely defined.[6] CoQ is an electron shuttle in the mitochondrial respiratory chain and a lipophilic antioxidant, contributing to energy production and redox homeostasis.[7] *COQ8B* is highly expressed in human aorta (see the GTEx Portal), but its function there has not been directly investigated.

The aortic smooth muscle cell (SMC) is central to the pathogenesis of TAA.[8] Aortic SMC disorganization and loss are characteristic of medial degeneration in TAA.[9] Mutations in SMC contractile genes cause familial TAA,[10] and SMC contractile gene expression is dysregulated across

[1]Department of Pediatrics, Indiana University School of Medicine, Indianapolis, IN 46202, USA; [2]Department of Medical and Molecular Genetics, Indiana University School of Medicine, Indianapolis, IN 46202, USA; [3]Department of Internal Medicine, McGovern Medical School, University of Texas Health Science Center at Houston, Houston, TX 77030, USA; [4]Department of Surgery, Indiana University School of Medicine, Indianapolis, IN 46202, USA; [5]Department of Pathology, Indiana University School of Medicine, Indianapolis, IN 46202, USA; [6]Divison of Cardiology, Cincinnati Children's Hospital Medical Center, Cincinnati, OH 45229, USA

*Correspondence: benjland@iu.edu

https://doi.org/10.1016/j.xhgg.2021.100057.

© 2021 The Author(s). This is an open access article under the CC BY license (http://creativecommons.org/licenses/by/4.0/).

different genetic etiologies.[11–14] Oxidative stress and abnormal mitochondrial respiration have been described in SMCs in different animal models of TAA,[15–20] and oxidative stress was observed in small series of human samples.[15,21] These findings require further investigation in larger numbers of human samples to understand the extent to which metabolic dysfunction in SMCs may be common to TAA. Recently, the common SNP rs3865452 (NM_024876.4[COQ8B]:c.521A>G [p.His174Arg]) was found to alter mitochondrial respiration in yeast, suggesting this SNP may impact diseases that are associated with metabolic dysfunction.[22] In this study, we investigated the expression and metabolic role of *COQ8B* in human proximal thoracic aortic SMCs. We interrogated proximal aortic tissues of individuals with TAA for mitochondrial and metabolic dysregulation. Finally, we tested the hypothesis that the *COQ8B* SNP rs3865452 was associated with TAA severity in two patient cohorts.

Subjects and methods

Study cohorts

Proximal thoracic aortic tissues

TAA tissue samples of the proximal aorta (aortic root or ascending aorta) were collected from individuals undergoing aortic surgery for TAA who consented and prospectively enrolled for study. Individuals with TAA or dissection secondary to traumatic injury were excluded. Control proximal aortic tissues were collected from individuals with normal aorta, including heart transplant recipients and organ donors. Participants were recruited in the Indiana University School of Medicine (IUSM) Cardiothoracic Surgery clinics or the National Donor Registry. Clinical data were collected through structured interviews by clinical research staff and review of the electronic medical record.

Longitudinal TAA study cohort

Young individuals who had TAA involving the proximal aorta or a genetic diagnosis predisposing to development of TAA were identified. Only individuals with at least two transthoracic echocardiograms performed before the age of 25 years were eligible, and echocardiograms after age 25 years were not utilized. Any echocardiograms performed after an aortic surgery were excluded. This cohort was prospectively enrolled in a cardiovascular genetics clinic at Cincinnati Children's Hospital Medical Center (CCHMC). Clinical data were collected by review of the electronic medical record. Clinical echocardiographic measurements of the aortic root and ascending aorta diameters were recorded for all individuals. The Z scores were generated for each measurement using body surface area-based nomograms published by the Pediatric Heart Network.[23]

Early-age onset of sporadic thoracic aortic dissection (ESTAD) cohort

Patients in this cohort had TAD before age 56 years and did not have an identified genetic cause on exome sequencing. A description of the characteristics of this cohort was previously published.[24]

Histological analysis of aortic tissues

Aortic tissue samples from aortic root or ascending aorta segments were fixed in 10% formalin solution immediately after explant. All histological studies were performed in conjunction with the IUSM clinical pathology laboratory. Tissues were embedded in paraffin and sectioned at 6 μm thickness. Sections were stained with hematoxylin and eosin (H&E) and Movat's pentachrome for analysis of medial degeneration using a standardized grading system.[25] None of the samples showed inflammatory aortitis. Adjacent sections were assayed for nitrotyrosine residues using anti-nitrotyrosine antibody (EMD Millipore, Burlington, MA, USA; AB5411). Processing included high pH antigen retrieval and 1:100 dilution. These were optimized using mouse cerebellum tissues treated with zymosan as per supplier's guidelines. Staining was performed using the Dako Flex+ system (Agilent). Incubation times were: 40/15/15/10 min for primary antibody/link/secondary antibody-labeled polymer/DAB and preceded by H_2O_2 block. Buffer washes were completed between each step. The intensity of nitrotyrosine staining in aortic tissues was graded semiquantitatively on a scale of 0 to 3. Scoring of medial degeneration characteristics and nitrotyrosine levels were performed by two readers (M.T.I. and B.J.L.) blinded to sample ID or group. The mean scores between the two readers were used for analysis. Nitrotyrosine intensity values of 1 or 1.5 were defined as mild, 2 or 2.5 as moderate, and 3 as severe.

Transmission electron microscopy (TEM) of aortic tissues

Aortic tissue specimens from the ascending aorta segment were fixed in 3% glutaraldehyde in phosphate buffer immediately after explant. The control and TAA specimens were consecutively acquired. Two of the controls were collected from organ donors, and one control was collected from a heart transplant recipient for ischemic cardiomyopathy. The specimens were post-fixed with 1% osmium tetroxide/phosphate buffer for 1 h. The specimens were then dehydrated through a series of graded ethyl alcohols, the infiltration process followed using acetone, with the final step being 1/2 100% acetone and 1/2 resin for 48 h. Embedding into blocks followed with 100% resin (Embed 812, Electron Microscopy Sciences, Hatfield, PA, USA). Blocks were sectioned after 12–18 h at 60°C for polymerization. Sections were viewed with light microscope for orientation and selection of medial layer. Sections were cut approximately 90 nm thick, placed on 200 mesh copper grids, uranyl acetate (UA) stained (saturated UA in 50% alcohol), and then viewed and imaged on a transmission electron microscope (Thermo Fisher Spirit, Hillsboro, OR, USA; Advanced Microscope Techniques, CCD camera, Danvers, MA, USA).

Investigators were blinded to sample ID and group throughout imaging and analysis. In the medial layer of aortic tissues, a mean 9 ± 2 SMCs were analyzed per sample. SMCs were examined from at least 5 randomly selected grid spaces per sample. Images for analysis were acquired at magnification of 890×, 11,000×, and 30,000×. Mitochondrial morphology assessments were performed using 30,000× images. The severity of ultrastructural defects in mitochondria were graded by a reader (B.J.L.) based on a previously developed scoring system.[26] Mitochondria were scored as follows: type 1: intact mitochondria with normal-appearing cristae; type 2: abnormal mitochondria with either swollen, irregular, or whorling cristae; type 3: mitochondria with discontinuous outer membrane or deficient cristae; or type 4: mitochondria with both swollen and deficient cristae or both discontinuous outer membrane and swollen cristae. Mean ultrastructural defect scores across all mitochondria studied were calculated for each sample, and these values were compared between TAA and control subjects.

Primary culture of aortic SMCs

Primary aortic SMCs were cultured from freshly explanted aortic root or ascending aorta tissues utilizing an explant outgrowth method. Intimal and adventitial layers of the aorta were dissected from the medial layer. Medial layer tissues were partitioned into 3- to 5-mm pieces and attached to Falcon T25 tissue flasks (Fisher) by remaining upright for 2 h in a humidified, 37°C, 5% CO_2 incubator. After 2 h, flasks were placed horizontally in the incubator with SMC growth medium composed of MCDB 131 (Gibco, Waltham, MA, USA) supplemented with 5% fetal bovine serum and the SmGM-2 Smooth Muscle SingleQuots Kit (Lonza, Basel, Switzerland) covering the tissue pieces. Growth medium was changed every 3 to 4 days until the SMCs covered at least 20% of the plate, which typically occurred by around 20 days after the initial tissue plating. SMCs were subsequently passaged, expanded, and routinely subcultured every 7 days.

siRNA knockdown of COQ8B

Small interfering RNA (siRNA) knockdown of *COQ8B* was performed in primary aortic SMCs. Knockdown was performed using Silencer Select Pre-Designed siRNA (Invitrogen, Waltham, MA, USA; s36676) targeting COQ8B/ADCK4 (siCOQ8B) or Silencer Select Negative Control No. 1 siRNA as control (siNeg). Transfection of siRNAs was performed using Lipofectamine RNAiMAX Transfection Reagent (Invitrogen) diluted in Opti-MEM Reduced Serum Medium (Gibco) as per protocol. SMCs were transfected at 50% to 70% confluency following the manufacturer's recommended protocol. siRNA experiments were performed in SMCs from healthy control subjects and from individuals with syndromic aortopathy (MFS or LDS).

RNA expression analysis

RNA was extracted from adherent SMCs using the RNeasy Mini Kit (QIAGEN, Germantown, MD, USA). RNA was quantified by spectrophotometry with NanoDrop 2000 and reverse transcribed with the High-Capacity cDNA Reverse Transcription Kit (Applied Biosystems, Waltham, MA, USA). Gene expression levels were measured by qRT-PCR using the Roche light cycler 96 RT-PCR system (Basel, Switzerland). Primers for target genes (Table S1) were designed with the Universal ProbeLibrary System Assay Design application (Roche) and synthesized by Integrated DNA Technologies (Coralville, IA, USA). Expression levels were indexed using the ΔΔCq method and *ACTB* as reference housekeeping gene.

Western blots

Whole-cell protein lysates were extracted using ice-cold 1× radioimmunoprecipitation assay (RIPA) buffer (Abcam, Cambridge, MA, USA; ab156034) supplemented with 1% Halt Protease and Phosphatase Inhibitor Cocktail (Thermo Scientific). Adherent SMCs were washed twice with ice-cold phosphate-buffered saline (PBS). RIPA was applied and then cells were scraped and collected into cold microcentrifuge tubes by pooling lysate of 3 wells of 6-well plate, nutated at 4°C for 60 min, centrifuged at 12,000 × *g* for 15 min, and then supernatant collected. Alternatively, the Cell Fractionation Kit (Abcam; ab109719) was used to extract subcellular compartment protein fractions (mitochondrial and cytosolic) from adherent SMCs following the manufacturer's protocol. Sample protein concentrations were measured using the Pierce BCA Protein assay (Thermo Scientific, Waltham, MA, USA).

For western blots, protein samples were combined with Laemmli Sample Buffer (Bio-Rad, Hercules, CA, USA), heated at 95°C for 5 min, and loaded into Novex Wedgewell 10% Tris-Glycine gels (Invitrogen). SDS-PAGE was performed with the Invitrogen Novex MiniCell system. Transfer to Immobilon Transfer Membrane (Millipore) was performed using the Mini-PROTEAN Tetra Cell system (Bio-Rad). The primary antibodies used for western blots were: ACTB (Abcam ab8227, dilution 1:5,000), CNN1 (Abcam ab46794; dilution 1:10,000), COXIV (Abcam ab202554, dilution 1:2,000), GAPDH (Abcam ab9485, dilution 1:5,000), ACTA2 (Abcam ab5694, dilution 1:1,000), and COQ8B (LSBio LS-C119206, dilution 1:500 and Atlas HPA028303, dilution 1:250). Antibodies were diluted in 5% blocking reagent (membrane blocking agent RPN2125V, GE Healthcare) in phosphate-buffered saline with 0.1% Tween 20. The membrane was blocked for 1 h at room temperature, incubated in primary antibody at 4°C overnight, and then incubated in secondary antibody for 1 h at room temperature using anti-rabbit IgG (Life Technologies A16110, dilution 1:10,000). The Precision Plus Protein WesternC Blotting Standards (Bio-Rad) and Precision Protein StrepTactin-HRP Conjugate (Bio-Rad) were used for chemiluminescent ladder. The colorimetric PageRule Prestained Protein Ladder was also used (Thermo Scientific). Clarity Western ECL Substrate (Bio-Rad) was applied to the membrane, and chemiluminescent images were acquired using the ChemiDoc Touch Imaging System and densitometry performed in Image Lab software (Bio-Rad).

Analysis of mitochondrial respiration in SMCs

Mitochondrial respiration was assayed using the Seahorse Bioscience XFp Analyzer (Agilent, Santa Clara, CA, USA) and the Mito Stress Test Kit. SMCs acquired from healthy aorta were transfected with siCOQ8B or siNeg. At 48 h after transfection, SMCs were passaged and seeded into an XFp plate at density of 30,000 SMCs per well in triplicate. All SMCs were visibly adherent by 4 h, at which time the Mito Stress Test was administered as per protocol. Briefly, the basal oxygen consumption rate (OCR) was measured before administering the complex V inhibitor oligomycin. Next, the uncoupling agent FCCP (carbonyl cyanide 4-(trifluoromethoxy)phenylhydrazone) creates uninhibited electron flow and measurement of maximal OCR. Rotenone and antimycin A abolish mitochondrial respiration by inhibiting complex I and III, allowing measurement of non-mitochondrial OCR. Maximal respiration was calculated by subtracting non-mitochondrial OCR from the maximal OCR measured after FCCP. Spare capacity was calculated by subtracting basal OCR from maximal OCR measured after FCCP. SMCs were imaged in the XFp plate before and after the assay, confirming similar levels of confluence and absence of cell loss during the assay. SMCs were lysed with RIPA upon completion and per-well protein quantities measured using the Pierce BCA Protein assay. The levels of OCR were indexed for protein quantity in analyses.

Immunoblot analysis for protein carbonylation in SMCs

Healthy control SMCs were transfected with siCOQ8B or siNeg in 6-well plates in triplicate wells per condition. At 48 h after transfection, whole-cell protein lysates were extracted using RIPA as described. Samples were immediately quantified for protein and prepared for carbonylation measurement using the OxyBlot Protein Oxidation Detection Kit (Sigma) per manufacturer's protocol. 6.4 μg protein were loaded per well and run on Novex Wedgewell 4%–12% Tris-Glycine mini gels (Invitrogen). Transfer membranes were incubated in the supplied primary antibody for 1 h at room

temperature. Post-imaging densitometry analysis was performed using Image Lab software.

Analysis of hydrogen peroxide levels in SMCs

Healthy control SMCs were transfected with siCOQ8B or siNeg in 12-well plates in triplicate wells for each condition. Approximately 48 h after transfection, the Amplex Red Hydrogen Peroxide/Peroxidase Assay Kit (Invitrogen) was utilized to measure levels of H_2O_2 based on the manufacturer's protocol and prior publication.[15] Immediately before the assay, phase-contrast images of SMCs were acquired using the IncuCyte Zoom Microplate Reader (Essen BioScience, Ann Arbor, MI, USA) and well confluency calculated using the IncuCyte software. After washing SMCs twice with warm HEPES-buffered Tyrode's solution (Alfa Aesar, Tewksbury, MA, USA), the Amplex Red reagent and horseradish peroxidase diluted in Tyrode's solution were added to each well. Cells were incubated for 30 min at 37°C in 5% CO_2. Extracellular fluid samples were transferred from each well to a 96-well plate in triplicate. Fluorescence levels were measured using the Synergy H4 Microplate Reader with Gen5 software. Readings were captured at 545 nm excitation and 590 nm emission. Fluorescence intensities were indexed for well confluency for analysis. Primary aortic SMCs from participants with TAA ($N = 2$) or normal control subjects ($N = 2$) without siRNA transfections were also assayed. In these experiments, SMCs were plated into 12-well plates in triplicate at 180,000 cells/well. Cells were visibly adherent after 4 h of plating and then assayed per protocol.

Assay for levels of malondialdehyde (MDA)

Aortic root SMCs from a 19-year-old male participant with MFS and TAA were plated into two T75 flasks (1,000,000 cells/flask) at passage 5 after initial plating of the tissue. siRNA transfection with siCOQ8B or siNeg was performed the following day. MDA levels were measured 48 h later according to protocol using the Lipid Peroxidation (MDA) Assay Kit (Abcam, Cambridge, MA, USA; ab118970). Cells were trypsinized 48 h after siRNA transfection and 1,000,000 cells utilized for assay per condition. Fluorescence signal was measured in triplicate using Synergy H4 Microplate Reader with Gen5 software with readings captured at 532 nm excitation and 553 nm emission. For positive control, cells from same SMC line (at passage 4) were exposed to 100 μM H_2O_2 for 24 h prior to assay. Positive control and siRNA experiments were replicated and yielded similar fold changes between experiments.

Comparison of COQ8B RNA and protein expression in primary aortic SMCs

At passage 2 or 3, SMCs were plated in Nunc Cell-Culture-treated 6-well plates (Thermo Scientific, Waltham, MA, USA) at density of 10,000 cells/cm^2. Growth media was changed on the subsequent day (day 2) and changed to low serum (0.5%) medium without growth factors on day 4 for 24 h before RNA and protein extraction.

Genotyping for rs3865452

Longitudinal TAA cohort

DNA was extracted from peripheral blood lymphocytes. Exome sequencing and variant calling was performed in the DNA Sequencing and Genotyping Core at CCHMC, as previously described.[4] Post-call variant analysis and filtering steps were performed using the SNP & Variation Suite (Golden Helix). Variants were filtered for genotype quality > 20, read depth > 10, and alt allele ratio thresholds of <0.15 for homozygous minor allele genotypes, 0.3 to 0.7 for heterozygous genotypes, and >0.85 for homozygous major allele genotypes. Principal component of population stratification was calculated using EIGENSOFT. The genotype of the rs3865452 SNP in *COQ8B* was identified from exome sequence analysis. Mean coverage of the SNP position 19:41211056 (assembly GRCh37) across studied samples was 43 ± 15 reads, ranging from 15 to 78 reads.

ESTAD cohort and healthy control subjects

Whole-exome sequencing of individuals with aortic dissection occurring before age 56 years was performed, as previously described.[24] Whole-exome sequencing data from healthy population control samples of European ancestry were acquired from the National Heart, Lung, and Blood Institute (NHLBI) Grand Opportunity Exome Sequencing Project (dbGap: phs000401) and University of Washington Human Genome Center (UW) (dbGap: phs000693). The rs3865452 genotype was obtained from the exome data. Exome sequencing of ESTAD cases and UW controls was performed contemporaneously at UW, and the variant calls in ESTAD cases and UW controls were generated together and aggregated into one v*cf.* file.

Cultured primary aortic SMCs

DNA was extracted from peripheral blood lymphocytes or directly from SMCs when blood was unavailable. The rs3865452 genotype was determined using TaqMan Genotyping Assay (Thermo Fisher) according to the manufacturer's protocol. The accuracy of the genotyping for rs3865452 was confirmed in a subset of samples that had exome sequencing.

Statistics

Nitrotyrosine intensity level was considered as an ordinal variable and compared between TAA and control aorta samples using the Cochran Armitage test. Multivariate ordinal regression analysis for nitrotyrosine level was performed to control for potential confounders such as age or other cardiovascular comorbidities. Association between age and mitochondrial ultrastructure defect score was tested by linear regression. RNA expression levels were compared between TAA and normal aorta SMCs and between siRNA conditions using the Student's t test (two-tailed distribution, two-sample unequal variance). The association between rs3865452 and longitudinal change in aortic Z scores was analyzed using a mixed model with a random variable adjusting for the correlations among the repeated measurements in the same individuals. The 1st and 2nd principal components derived from exomes were included to adjust for population stratification. The statistical software utilized was SAS and JMP (Cary, NC, USA).

Study approval

This study was approved by the Institutional Review Boards at Indiana University School of Medicine, Cincinnati Children's Hospital Medical Center, and University of Texas Health Science Center at Houston. Written informed consent was received from participants prior to inclusion in the study. Tissue organ donors provided consent for research. The procedures followed were in accordance with the ethical standards of the responsible committee on human experimentation (institutional and national), and proper informed consent was obtained.

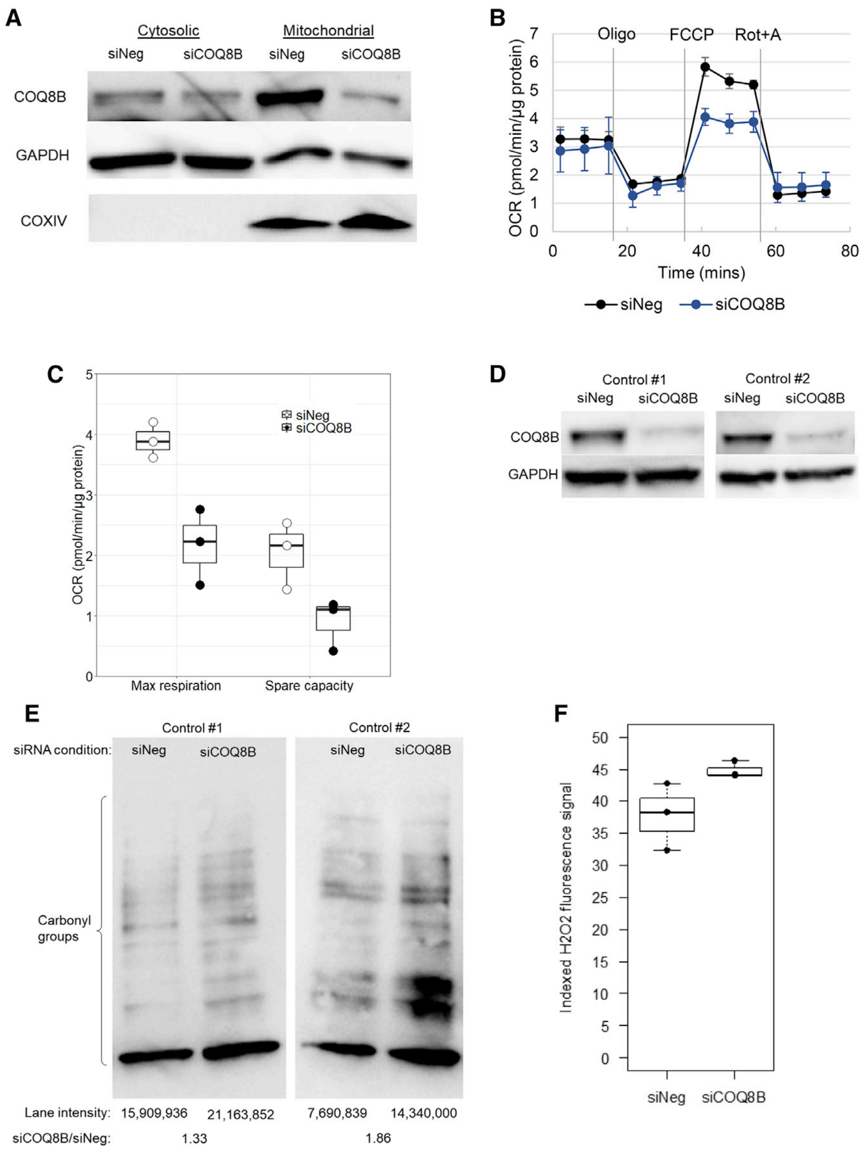

Figure 1. Loss of mitochondrial COQ8B impairs aortic SMC mitochondrial respiration and increases oxidative stress
(A) Western blot for COQ8B in cytosolic and mitochondrial fractions of healthy control aortic SMCs 48 h post-siRNA transfection targeting COQ8B (siCOQ8B) or negative control siRNA (siNeg). COXIV is a mitochondrial marker and GADPH a cytosolic marker.
(B) Oxygen consumption rate (OCR) measured during sequential administration of oligomycin (Oligo), oxidative phosphorylation uncoupler (FCCP), and rotenone (Rot) and antimycin A (Ant) in SMCs transfected with siCOQ8B or siNeg.
(C) Maximal respiration and spare capacity calculated from the OCR measurements shown in (B).
(D) Western blot for COQ8B in total cellular protein samples in siCOQ8B and siNeg SMCs. siRNA transfections were performed in SMCs from two separate control participants (Control #1 and Control #2).
(E) Protein carbonylation levels measured using Oxyblot in samples of (D). Chemiluminescent signal intensity is shown below each lane (Lane intensity) and used to calculate siCOQ8B/siNeg ratios.
(F) Levels of H_2O_2 production in aortic SMCs measured by Amplex Red in siCOQ8B and siNeg SMCs. Emitted fluorescent signal (590 nm) was indexed for cell confluency.
Ant, antimycin A; FCCP, carbonyl cyanide 4-(trifluoromethoxy)phenylhydrazone; Oligo, oligomycin; OCR, oxygen consumption rate; Rot, rotenone. *$p < 0.05$.

Results

Loss of COQ8B in mitochondria of aortic SMCs leads to metabolic dysregulation and altered contractile gene expression

To investigate *COQ8B* in human aortic SMCs, primary SMCs cultured from healthy donor ascending aorta tissues were transfected with siRNA targeting *COQ8B* (siCOQ8B) or non-targeting siNeg. COQ8B protein was abundant in the mitochondrial fraction in controls, while siCOQ8B transfection led to significant decrease in mitochondrial COQ8B (Figure 1A). The mitochondrial bioenergetic parameters of maximal respiration and spare respiratory capacity were decreased by approximately 50% in siCOQ8B SMCs compared with siNeg (Figures 1B and 1C). Additionally, total cellular protein lysates from siCOQ8B SMCs displayed a 33% to 86% increase in protein carbonylation compared with siNeg, consistent with increased oxidative stress (Figures 1D and 1E). This finding was replicated twice more in separate siRNA transfection experiments to confirm reproducibility (Figure S1A), and the Oxyblot assay sensitivity was verified by dose-dependent increases in carbonylation in SMCs administered incremental dosages of the reactive oxygen species (ROS) H_2O_2 (Figure S1B). Knockdown of COQ8B protein in siCOQ8B SMCs was further confirmed using a second COQ8B antibody (Figure S2). To further investigate oxidative stress, endogenous H_2O_2 levels were compared between siCOQ8B and siNeg SMCs using the Amplex Red assay in live adherent cells. H_2O_2 levels were increased by approximately 20% in siCOQ8B SMCs (Figure 1F). Throughout these experiments, siCOQ8B transfection did not result in significant cell loss. These results indicate that loss of *COQ8B* impairs mitochondrial respiration and increases oxidative stress in aortic SMCs without having a significant effect on cell survival.

Smooth muscle contractile gene expression is frequently dysregulated in TAA.[11–14] We found that *COQ8B* knockdown was associated with significantly altered transcription of the SMC contractile gene *CNN1* (CNN1 [MIM: 600806]) and *MYOCD* (MYOCD [MIM: 606127]), a master regulator of SMC contractile gene expression (Figure 2A).

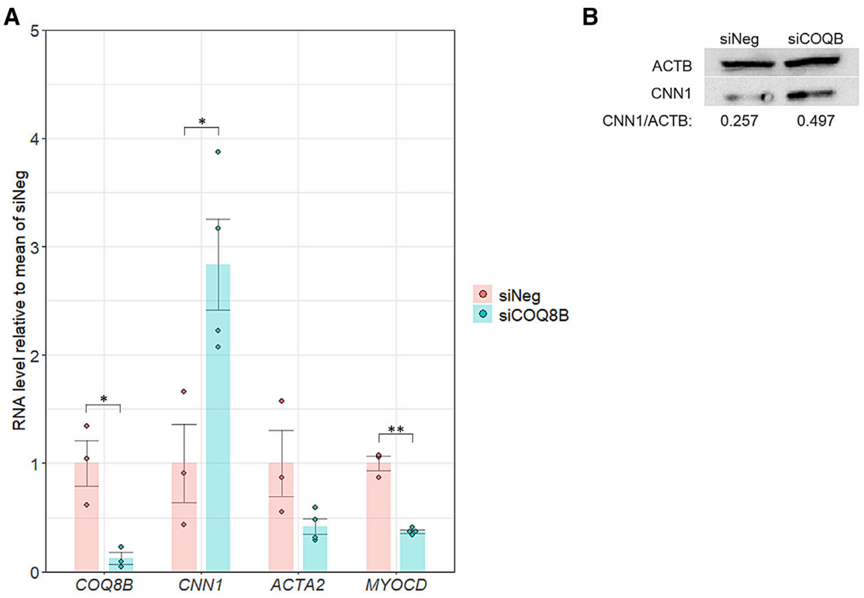

Figure 2. Expression levels of SMC contractile genes are altered with knockdown of *COQ8B*
(A) RNA levels of *COQ8B* and genes important for SMC contraction measured by qRT-PCR 48 h after siRNA knockdown of COQ8B (siCOQ8B) or negative control siRNA (siNeg). Each point represents a sample extracted from an independently transfected well. Target gene RNA levels are adjusted for *ACTB* and displayed relative to mean of siNeg SMCs. Shaded bars show means ± standard error. Levels were compared between conditions using Student's t test. $^*p < 0.05$; $^{**}p < 0.01$.
(B) Western blot of calponin-1 (CNN1) levels between siCOQ8B and siNeg SMCs in protein extracted concurrently with RNA samples. Ratios of chemiluminescent signal intensity between CNN1 and loading control ACTB are shown.

Protein levels of the *CNN1* product calponin-1 were also increased in siCOQ8B SMCs (Figure 2B). RNA levels of the contractile gene *ACTA2* (ACTA2 [MIM: 102620]) were decreased in siCOQ8B SMCs but did not reach statistical significance ($p = 0.19$). The protein levels of smooth muscle alpha actin (*ACTA2* product) were not different between siCOQ8B and siNeg SMCs. Taken together, these results indicate that loss of COQ8B in human aortic SMCs is associated with dysregulated contractile gene expression, a common pathological feature of TAA, along with metabolic dysfunction.

Oxidative stress, mitochondrial defects, and decreased COQ8B expression in human TAA

In order to investigate metabolic dysregulation in human TAA broadly, aortic root and ascending aorta tissues were acquired from individuals undergoing aortic repair surgery for TAA ($N = 74$) or control subjects ($N = 23$) (Table 1). The mean maximum aortic root or ascending aorta diameter among TAA individuals was 5.2 ± 0.6 cm, consistent with severe proximal aortic dilation. Medial degeneration was observed in TAA samples, using established histological analysis of fixed tissues.[25] The intensity of staining for nitrotyrosine residues, a marker of oxidative stress, was significantly increased in TAA tissues compared with healthy controls ($p = 0.03$) (Figures 3A and 3B). Nitrotyrosine intensity was associated with TAA independent of hypertension, dyslipidemia, sex, and age in multivariable regression ($p = 0.007$). In addition, transmission electron microscopy of aortic tissues identified markedly severe mitochondrial cristae defects in six of seven TAA samples compared with controls (Figures 3C and 3D). Overall, approximately 35% of all mitochondria in TAA samples had the most severe defect score of 4, compared with <5% of mitochondria in controls (Figure S3). Mild cristae changes that were observed in the mitochondria of controls may be technical or subclinical. The clinical characteristics of participants studied with TEM are provided in Table S2. Control subjects were on average younger than affected individuals (36 ± 25 versus 58 ± 10 years), but age and the severity of ultrastructural defects were not correlated ($p = 0.33$). Along with the *in situ* evidence of metabolic dysfunction in TAA tissue, in culture the levels of H_2O_2 were approximately 50% higher in primary aortic SMCs cultured from TAA tissues compared with controls (Figure 3E). Thus, oxidative stress and mitochondrial defects were prevalent in a large, etiologically heterogeneous series of human TAA tissues.

Endogenous RNA levels of *COQ8B* were measured in aortic SMCs cultured from participants with TAA ($N = 7$) or control subjects ($N = 4$) (participant characteristics in Table S3). In RNA samples extracted at second cell passage in triplicate, *COQ8B* levels were significantly decreased in TAA compared with controls on average by approximately 50% (Figure 3F; Figure S4). Taken together, TAA was associated with increased oxidative stress and mitochondrial defects and decreased *COQ8B* expression. These findings correlate with the observed effects of *COQ8B* knockdown in aortic SMCs.

Decreased COQ8B leads to increased oxidative stress and altered contractile gene expression in syndromic TAA

To investigate the effects of decreased COQ8B in TAA, aortic SMCs from participants with TAA and MFS were transfected with siCOQ8B or siNeg. As expected, mitochondrial levels of COQ8B protein decreased after siCOQ8B transfection (Figure S5A). Again, increased protein carbonylation was observed in siCOQ8B SMCs (Figure 4A), although the relative increase was less pronounced than was observed with *COQ8B* knockdown in healthy control SMCs (Figure 1E). In addition, siCOQ8B transfection in

Table 1. Demographic and clinical characteristics of cohort with aortic histological studies.

	Control (N = 23)	TAA (N = 74)
Age (mean ± standard deviation), years	38.9 ± 19.4	58.8 ± 13.8
**Sex, n (%)**		
Male	18 (78)	62 (84)
Female	5 (22)	12 (16)
**Race, n (%)**		
White	14 (100)	66 (89)
Black	0	7 (9)
Asian	0	1 (1)
Family history of TAA, n (%)	0	9 (12)
Thoracic aortic dissection, n (%)	0	9 (12)
**Aortic valve morphology, n (%)**		
Tricuspid	22 (96)	41 (55)
Bicuspid	1 (4)	33 (45)[a]
**Aortic segment studied, n (%)**		
Root	7 (30)	10 (14)
Ascending aorta	16 (70)	62 (84)
Root versus ascending not specified	0	2 (3)
Hypertension, n (%)	6 (26)	52 (71)
Dyslipidemia, n (%)	1 (4)	36 (49)
Type 2 diabetes, n (%)	2 (9)	13 (16)
Coronary artery disease, n (%)	3 (13)	26 (35)
Marfan syndrome, n (%)	0	2 (3)

% of available. TAA, thoracic aortic aneurysm.
[a]Includes one case with unicuspid aortic valve.

MFS SMCs led to an approximately 40% increase in levels of malondialdehyde (Figure 4B), which is a product of lipid peroxidation and companion marker for oxidative stress. For reference, SMCs exposed to 100 μM H_2O_2 for 24 h had 13% to 25% increase in MDA levels (data not shown). *COQ8B* knockdown also altered contractile gene expression in SMCs from participants with MFS (Figure 4C) and LDS (Figure 4D). This included marked increase in the levels of *CNN1* RNA and the expressed protein calponin-1 (Figure 4E). Overall, these findings recapitulate the effects of COQ8B loss that were observed in healthy control SMCs. This indicates that reduced COQ8B level is deleterious for SMC metabolism and contractile gene expression in the setting of TAA.

A common COQ8B SNP (rs3865452) is associated with decreased rate of aortic root dilation in young patients

Previously, yeast expressing human *COQ8B* with the common SNP rs3865452 (c.521A>G [p.His174Arg]) were found to have increased COQ8B protein levels and respiration compared with the reference allele.[22] Here, we investigated rs3865452 in 48 unrelated young patients who had TAA or genetic diagnosis predisposing to TAA and longitudinal echocardiographic follow-up (Table 2; Table S4). Aortic root and ascending aorta diameters were acquired from a mean 6.8 ± 4.0 echocardiograms per individual, which were performed over a span of 5.3 ± 4.1 years. Overall, this duration of follow-up is relatively long compared with a major drug trial in young individuals with TAA that utilized change in aortic *Z* score as the primary outcome.[27] The mean age at first echocardiogram was 10.0 ± 5.0 years. The mean aortic root *Z* score at the first echocardiogram was 3.7 ± 1.8, while the mean *Z* score was 1.7 ± 2.1 for the ascending aorta segment. This indicates that the cohort overall had more severe involvement of the aortic root than ascending aorta. A mixed-model analysis of longitudinal aortic root *Z* score values identified inverse association between aortic root *Z* score and the interaction between rs3865452 and time ($p = 0.0003$) (Table 3). This indicates that the rs3865452 SNP was significantly associated with a decreased rate of change in aortic root *Z* score. The estimated effect of rs3865452 on the longitudinal rate of aortic root dilation was −0.14 ± 0.04 *Z* score/year per G allele (Figure 5). Including sex and syndromic diagnosis as covariates, the association between aortic root *Z* score and the rs3865452 × time interaction term was significant ($p = 0.03$). In post hoc review of medical therapy history, all 13 affected individuals homozygous for the reference allele (AA) and 23 of 25 heterozygous for rs3865452 (AG) were treated medically for TAA, while only 5 of 10 individuals homozygous for rs3865452 (GG) were on medical therapy. Unlike the significant finding for aortic root, the rs3865452 SNP was not significant in a mixed-model analysis for the longitudinal ascending aorta *Z* scores. Overall, these results suggest that rs3865452 has a protective effect against progressive aortic root dilation in young TAA individuals.

Frequency of rs3865452 in early TAD cases

Separately, we investigated the frequencies of rs3865452 genotypes in a distinct exome analysis of 230 individuals of European ancestry (EA) who had ESTAD. This disease cohort is composed of individuals with severe disease, who had a TAD before the age of 56 years, as was previously described.[24] In this severe phenotype cohort, the proportions of AG and GG genotypes were lower, and AA genotypes higher, than observed in ancestry-matched controls from the NHLBI Grand Opportunity Exome Sequencing Project (ESP) (N = 4,300) or in 282 additional EA controls sequenced in the same lab (UW) as ESTAD cases (Figure 6A). Accordingly, the allele frequency of rs3865452 was lower in ESTAD cases (0.446) than ESP (0.494) or UW controls (0.5) (Figure 6B). This finding in a cohort of individuals with the severe phenotype of TAD

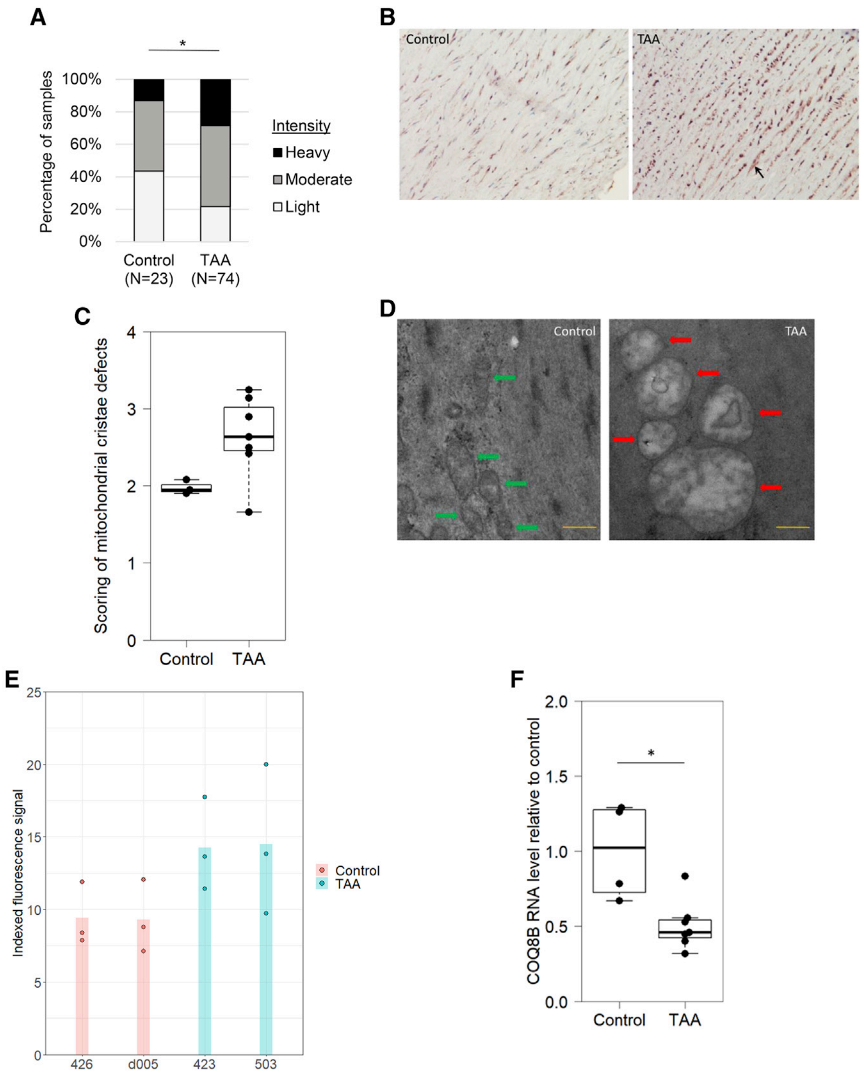

Figure 3. Oxidative stress and mitochondrial damage in the proximal aorta in TAA
(A) Semiquantitative scoring of the intensity levels of intracellular anti-nitrotyrosine staining in the aortic medial layer between TAA and controls. Groups were compared using Cochran-Armitage test.
(B) Example of heavy anti-nitrotyrosine staining intensity in the ascending aortic medial layer in TAA compared with control aorta. Blue staining labels nuclei and brown staining labels nitrotyrosine. Black arrow indicates a cell with heavy staining.
(C) Scoring of mitochondrial cristae defects between TAA (N = 7 individuals) and control (N = 3 individuals) aortic tissues imaged by transmission electron microscopy. Each point represents the mean score per individual. A median 79 (interquartile range [IQR], 55 to 117) mitochondria were analyzed per individual.
(D) Examples of normal mitochondria in controls (green arrows) and enlarged mitochondria with abnormal cristae in TAA (red arrows). Yellow bar indicates 250 nm.
(E) Levels of H_2O_2 in cultured aortic SMCs from TAA (N = 2 individuals) and control aorta (N = 2 individuals) measured by Amplex Red fluorescence intensity indexed for cell confluency. Each point is the result of an independent well, and bars indicate mean for each individual.
(F) RNA levels of *COQ8B* between TAA (N = 7 cases) and controls (N = 4 individuals) in cultured proximal aortic SMCs. *COQ8B* RNA levels were adjusted for *ACTB* and are displayed relative to mean of control SMCs. Each point corresponds to the mean of three independent wells of SMCs. Replicate values are shown in Figure S4. TAA and control groups were compared using a Student's t test.
*$p < 0.05$.

provides further evidence that rs3865452 is associated with relative protection in TAA.

rs3865452 and endogenous COQ8B protein levels in TAA

To investigate the biochemical role of rs3865452 in TAA, whole-cell protein lysates were extracted from primary aortic SMCs at early passage. The levels of COQ8B were compared between 6 participants with TAA who were homozygous for rs3865452 (GG) versus 6 who were homozygous for reference allele (AA) using western blots. This was accomplished using two western blots, each with 3 participants per homozygous group. The samples from GG participants contained higher levels of COQ8B protein compared with AA samples (Figure 7; Figure S6). Meanwhile, there was no significant difference in *COQ8B* RNA levels between homozygous groups in samples extracted in parallel with the protein extractions (Figure S7). The participants studied in Figure 3F were also genotyped for rs3865452 post hoc, and there was no difference in *COQ8B* RNA levels between genotypes in these samples either. The findings indicate that the rs3865452 SNP leads to increased COQ8B protein levels in human TAA.

Discussion

COQ8B has an important but incompletely defined role in the biosynthesis of CoQ, and recessive mutations are associated with a nephropathy (NPHS9 [MIM: 615573]) that typically manifests in adolescence.[6] In the present study, loss of COQ8B in human aortic SMCs led to decreased mitochondrial respiration and increased oxidative stress. This indicates that metabolism of aortic SMCs is susceptible to COQ8B loss, which is congruent with effects seen with its loss in other tissues, including podocytes[28] and dermal fibroblasts.[6] Consistent with this role, we identify mitochondrial localization of COQ8B in human aortic SMCs for the first time. We identified increased oxidative

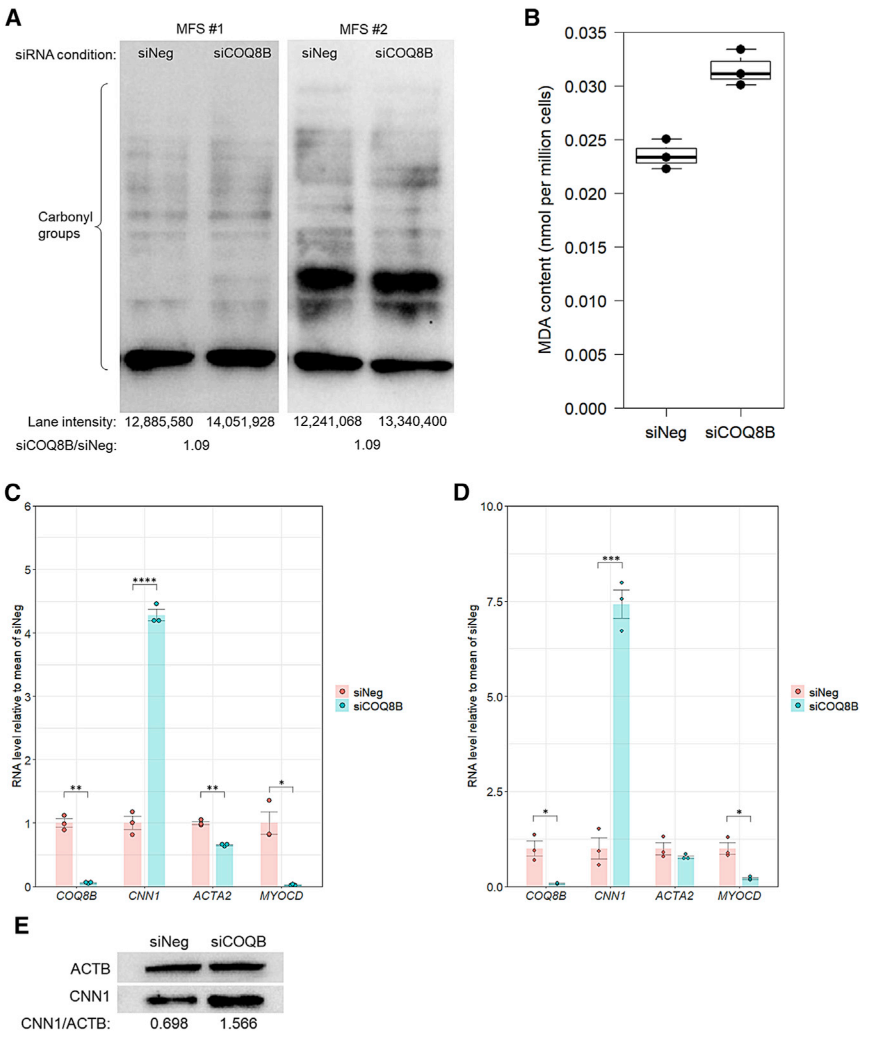

Figure 4. Knockdown of COQ8B in aortic SMCs from individuals with syndromic TAA leads to increased oxidative stress and altered contractile gene expression

(A) Protein carbonylation levels in aortic SMCs from two individuals with Marfan syndrome (MFS #1 and #2) transfected with siCOQ8B or siNeg. Protein levels of COQ8B were decreased in siCOQ8B (Figure S5B).

(B) Levels of the lipid peroxidation byproduct malondialdehyde (MDA) in MFS aortic SMCs transfected with siCOQ8B versus siNeg. Each sample was measured in triplicate, as shown by the points.

(C and D) RNA levels of *COQ8B* and SMC contractile genes measured in MFS (C) or LDS (D) aortic SMCs that were transfected with siCOQ8B or siNeg. Each point represents RNA extracted from an independently transfected well. Target gene RNA levels are adjusted for *ACTB* and displayed relative to mean of siNeg SMCs. Shaded bars show means ± standard error. Levels were compared between conditions using Student's t test. *$p < 0.05$; **$p < 0.01$; ***$p < 0.001$; ****$p < 0.0001$.

(E) Western blot of CNN1 levels between siCOQ8B and siNeg SMCs in lysates extracted concurrently with RNA samples in LDS. Ratios of chemiluminescent signal intensity between CNN1 and loading control ACTB are shown.

stress in the largest reported human TAA tissue series to date[21,29] and distinctively performed semiquantitative analysis of the ultrastructure of mitochondrial cristae, revealing novel evidence that cristae defects were common in human TAA. The effects of COQ8B loss in aortic SMCs were consistent with this pathology, and indeed *COQ8B* transcription was found to be decreased in TAA. Taken together with prior evidence for oxidative stress and mitochondrial dysfunction in multiple different genetic TAA mouse models,[15–18] the findings indicate that metabolic dysfunction is common to TAA in diverse genetic and developmental contexts. In this emerging paradigm of TAA pathogenesis, perturbations in metabolic pathway components, such as *COQ8B*, have the potential to impact TAA broadly.

Yeast experiments previously revealed that the human SNP rs3865452 increased mitochondrial COQ8B protein levels and thereby augmented mitochondrial respiration in comparison with the reference allele.[22] We report here new evidence in two separate cohorts, one with early-onset aortic dissection and one with longitudinal follow-up echocardiographic data in pediatric patients, that rs3865452 was associated with protection against TAA progression and dissection. We also observed that the SNP was associated with increased COQ8B protein levels in aortic SMCs in TAA. This is the first evidence for a biochemical effect of rs3865452 in human tissues, which compellingly recapitulates Vazquez Fonseca et al.'s[22] original observation in yeast. Importantly, we have observed this effect using endogenous protein levels in primary cells obtained directly from the diseased aortic tissues, which significantly increases the relevance to human TAA. The SNP was not associated with changes in *COQ8B* RNA levels, indicating the protein level effects may be post-transcriptional, as was suggested previously.[22] The evidence together indicates that increases in COQ8B protein levels associated with rs3865452 may ameliorate pathological metabolic dysregulation in TAA, which in this study included transcriptional downregulation of *COQ8B*. The physiological benefits of higher COQ8B levels in TAA may be to preserve mitochondrial respiration or antioxidant capacity via its role in CoQ production. Thus, rs3865452 may oppose pathological downregulation of *COQ8B* transcription, alleviate the metabolic stress associated with TAA, and thereby contribute to a less-severe TAA phenotype.

In the present study, the metabolic changes associated with knockdown of *COQ8B* in aortic SMCs were not

Table 2. Characteristics of 48 young TAA individuals with longitudinal aortic *Z* score measurements.

Characteristic	Number (%)
Sex	
Male	39 (81)
Female	9 (19)
Race	
White	45 (94)
Black or African American	3 (6)
Ethnicity	
Non-Hispanic	47 (98)
Hispanic	1 (2)
Syndromic classification	
Nonsyndromic, non-familial	22 (46)
Nonsyndromic, familial	3 (6)
Marfan syndrome	19 (40)
Loeys-Dietz syndrome	4 (8)
Bicuspid aortic valve	14 (29)
Hypertension	0 (0)
Prescribed medical therapy for TAA[a]	41 (87)
Aortic replacement surgery	3 (6)

TAA, thoracic aortic aneurysm.
[a]Data not available for one individual.

Table 3. Results of mixed-model analysis for the association between the *COQ8B* SNP rs3865452 and longitudinal aortic root *Z* scores

Effect	β ± SE	p value
Intercept	1.66 ± 0.81	0.06
PC1	−61.61 ± 28.48	0.03
PC2	116.58 ± 37.49	0.002
rs3865452	−0.01 ± 0.21	0.95
Time	0.11 ± 0.04	0.008
Time × rs3865452	*−0.14 ± 0.04*	*0.0003*

Italics indicate the significant association between aortic root *Z* score and the interaction of time × rs3865452. PC1, principal component 1; PC2, principal component 2; SE, standard error.

sufficient to trigger cell death. This is consistent with prior evidence in animal and cellular models of *COQ8B* loss. There is likely redundancy or compensatory mechanisms available when *COQ8B* is altered.[6,28] It is therefore plausible for the genotype of rs3865452 to alter COQ8B protein significantly but still be frequent in healthy populations. Increases in cellular metabolic stress initiated by a primary causative TAA gene mutation, abnormal vascular development, or exposure to hemodynamic stress may be a prerequisite for the physiological and clinical impact of the SNP to manifest in TAA. It is uncommon for individuals with TAA to suffer a TAD before reaching reproductive age, so selection pressure related to TAA is unlikely to influence the population frequency of rs3865452. It has, however, been noted that the herein proposed protective rs3865452 SNP is the reference (wild-type) allele in most mammalian species, suggesting a possible evolutionary advantage in ancestral species.[22]

In addition to metabolic changes, loss of COQ8B in SMCs was associated with altered expression of genes that are important for SMC contraction. This included significantly increased expression of *CNN1*, which is a regulator of SMC contractility. Increased *CNN1* in aortic tissues has been observed in TAA,[14,30,31] and increased calponin serum level has been proposed as a possible biomarker for TAD.[32] These data support that increase in *CNN1* has negative effects on aortic SMC homeostasis. *COQ8B* knockdown led to an impairment in mitochondrial bioenergetics. Because mitochondria are important for SMC contraction, this alteration could lead to a dysregulated contractile gene expression. Understanding precisely how *COQ8B* loss may impact the expression of *CNN1* and other SMC contractile genes warrants further investigation. In general, the interaction between SMC energy metabolism and intracellular biomechanics in TAA is not well understood.

Clinically, the early diagnosis of TAA provides the opportunity to initiate medical therapy, counsel on safe physical activities, and monitor for latent progression with imaging. However, these interventions may at the same time have adverse effects on physical and psychosocial health, as well as financial costs. Variable expressivity and incomplete penetrance are common in syndromic and nonsyndromic TAA, and therefore a genetic diagnosis alone is an imperfect predictor of risk. Currently, there is a paucity of clinical factors that predict the risk for progressive TAA and stratify management accordingly. Moreover, while guidelines for timing of surgical aortic repair have been developed, TAD occurs at variable aortic sizes including below established aortic diameter thresholds for prophylactic surgery.[33] Identifying additional genetic factors that modify risk for TAA progression or early TAD is a key next step to improve clinical care. Genetic analyses in this study included syndromic and nonsyndromic cases, and the results suggest that rs3865452 may influence TAA severity across different clinical subtypes. The frequent nature of metabolic abnormalities identified across TAA tissues and functional effects of COQ8B loss on metabolism in this study supports this proposition. The results of our longitudinal study of young patients suggests that rs3865452 has an impact on pathogenesis before late stages of disease. Indeed, recent data from mouse models identify metabolic dysregulation early in pathogenesis,[16,18] and markers of oxidative stress have been observed in blood samples of young individuals with MFS.[34] Ultimately, this study's findings may lead to use of rs3865452 genotyping to more precisely tailor clinical management starting at young ages and across subpopulations of TAA.

One limitation of this study is that genetic analyses of rs3865452 included retrospective data. Prospective studies

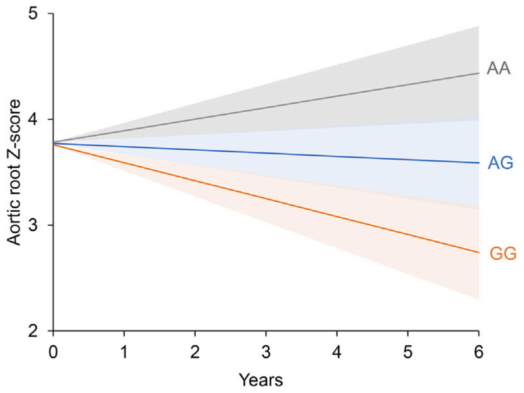

Figure 5. Model of the estimated effect of the *COQ8B* SNP rs3865452:A>G on longitudinal aortic root *Z* scores
Line slopes are the estimated effects of the rs3865452 genotypes derived from the mixed model analysis, and 95% confidence intervals are displayed as shaded bands.

are indicated. In longitudinal analysis, there were fewer individuals with dilation of the ascending aorta than the root. Future cohorts including more young individuals with ascending aorta dilation should be pursued. The impact of rs3865452 on COQ8B protein warrants further study in greater numbers of samples, including in other age categories and in healthy control samples. While significant associations between rs3865452 and TAA severity were identified, the genotype does not fully account for variation in TAA severity. For example, some ESTAD cases were homozygous for rs3865452, which means that this genotype does not completely protect individuals from TAD. The sample sizes in this study were underpowered to perform genome-wide study. Further studies are needed to identify the additional factors that modulate TAA severity. There were age differences between TAA and control tissue samples. While we did not find age to be significantly associated with severity of mitochondrial defects or oxidative stress in this study, future studies could more closely match cases and controls for age, because there is evidence that mitochondrial changes occur with aging. The majority of included study participants were of European ancestry, due in part to regional demographics and to limit effects of population stratification. Future larger studies with broader racial and ethnic diversity are required.

In summary, this study makes important strides toward understanding the genetic factors controlling TAA progression. We identify a SNP in *COQ8B* as a novel genetic factor and build evidence in human tissues that *COQ8B* may impact the mechanism of mitochondrial respiration and oxidative stress in TAA. Ultimately, testing for the *COQ8B* SNP in the clinical setting may lead to improved risk stratification and outcomes.

Data and code availability

The datasets supporting the current study are deposited in a public repository, ESTAD (dbGap: phs000401), and University of Washington Human Genome Center (UW) (dbGap: phs000693). Exome datasets not generated with federal funding have not been deposited in a public repository because of ongoing use for separate analyses but are available from the corresponding author on request.

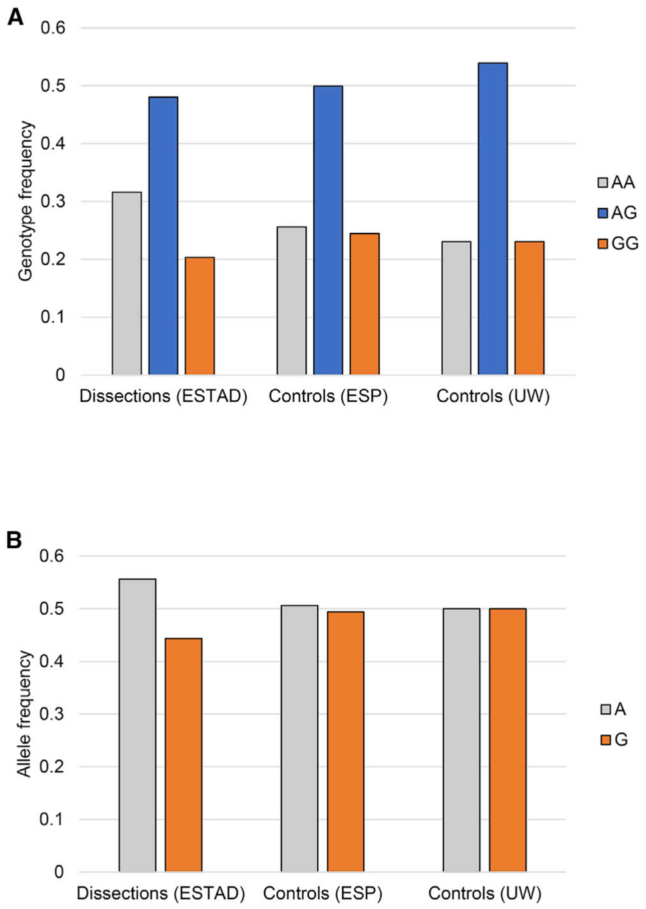

Figure 6. Frequency of the *COQ8B* SNP rs3865452:A>G in thoracic aortic dissection (TAD) versus controls
(A) Distributions of rs3865452 genotypes among early-age onset of sporadic TAD cases (ESTAD, N = 230) and ancestry-matched controls from the NHLBI GO Exome Sequencing project (ESP, N = 4,300) or University of Washington Center for Mendelian Genomics (UW, N = 282).
(B) Allele frequency of rs3865452 between cases (ESTAD) and two control populations (ESP and UW).

Supplemental information

Supplemental information can be found online at https://doi.org/10.1016/j.xhgg.2021.100057.

Acknowledgments

We acknowledge the contributions of Lindsey Elmore, Lauren Frasier, and Adam Oldham in clinical study coordination and data collection at IUSM. Indiana Clinical Translational Science Initiative core facilities contributing to this work were the Electron Microscopy Center (Caroline Miller) and Clinical and Translational Support Laboratory (Robert Orr). The IUSM Department of Pathology Immunohistochemistry Core performed tissue histology (Dr. Constance Temm). B.J.L. received support from NIH

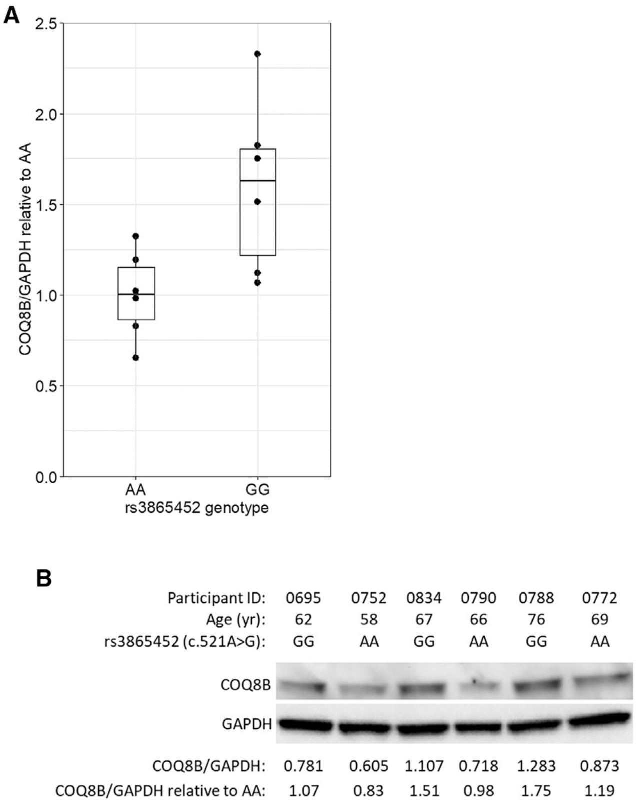

Figure 7. The *COQ8B* SNP rs3865452:A>G is associated with higher levels of COQ8B protein
(A) Western blots for COQ8B and GAPDH (loading control) were performed in whole-cell lysate of proximal aortic SMCs from a total 12 individuals with TAA who were either homozygous for rs3865452 (GG; N = 6) or homozygous for reference allele (AA; N = 6). COQ8B/GAPDH intensity values are shown relative to the mean of AA samples.
(B) Representative western blot that contains 6 of the samples included in (A), all collected at passage 2. The ratios of chemiluminescent signal intensity between COQ8B and GAPDH bands are shown for each sample and are also shown relative to the mean for AA samples in the blot.

awards K12HD068371 and K23HL141667 (Bethesda, MD), a Marfan Foundation Early Investigator Grant (Port Washington, NY), and the IUSM Strategic Research Initiative (Indianapolis, IN). S.M.W. received support from the IUSM Physician-Scientist Initiative and the Strategic Research Initiative (Indianapolis, IN). D.M.M. received support from NIH grant R01HL109942 (Bethesda, MD) and the John Ritter Foundation for Aortic Health (Los Angeles, CA, USA).

Declaration of interests

The authors declare no competing interests.

Received: June 21, 2021
Accepted: August 6, 2021

Web resources

EIGENSOFT, https://www.hsph.harvard.edu/alkes-price/software/
GTEx, https://gtexportal.org/home/index.html
OMIM, https://www.omim.org/

References

1. Kochanek, K.D., Murphy, S.L., and Xu, J. (2015). Deaths: Final Data for 2011. Natl. Vital Stat. Rep. *63*, 1–120.
2. Hiratzka, L.F., Bakris, G.L., Beckman, J.A., Bersin, R.M., Carr, V.F., Casey, D.E., Jr., Eagle, K.A., Hermann, L.K., Isselbacher, E.M., Kazerooni, E.A., et al.; American College of Cardiology Foundation/American Heart Association Task Force on Practice Guidelines; American Association for Thoracic Surgery; American College of Radiology; American Stroke Association; Society of Cardiovascular Anesthesiologists; Society for Cardiovascular Angiography and Interventions; Society of Interventional Radiology; Society of Thoracic Surgeons; and Society for Vascular Medicine (2010). 2010 ACCF/AHA/AATS/ACR/ASA/SCA/SCAI/SIR/STS/SVM guidelines for the diagnosis and management of patients with Thoracic Aortic Disease: a report of the American College of Cardiology Foundation/American Heart Association Task Force on Practice Guidelines, American Association for Thoracic Surgery, American College of Radiology, American Stroke Association, Society of Cardiovascular Anesthesiologists, Society for Cardiovascular Angiography and Interventions, Society of Interventional Radiology, Society of Thoracic Surgeons, and Society for Vascular Medicine. Circulation *121*, e266–e369.
3. Wheeler, A.P., Yang, Z., Cordes, T.M., Markham, L.W., and Landis, B.J. (2021). Characterization of the Rate of Aortic Dilation in Young Patients with Thoracic Aortic Aneurysm. Pediatr. Cardiol. *42*, 148–157.
4. Landis, B.J., Schubert, J.A., Lai, D., Jegga, A.G., Shikany, A.R., Foroud, T., Ware, S.M., and Hinton, R.B. (2017). Exome Sequencing Identifies Candidate Genetic Modifiers of Syndromic and Familial Thoracic Aortic Aneurysm Severity. J. Cardiovasc. Transl. Res. *10*, 423–432.
5. Verma, S., and Siu, S.C. (2014). Aortic dilatation in patients with bicuspid aortic valve. N. Engl. J. Med. *370*, 1920–1929.
6. Ashraf, S., Gee, H.Y., Woerner, S., Xie, L.X., Vega-Warner, V., Lovric, S., Fang, H., Song, X., Cattran, D.C., Avila-Casado, C., et al. (2013). ADCK4 mutations promote steroid-resistant nephrotic syndrome through CoQ10 biosynthesis disruption. J. Clin. Invest. *123*, 5179–5189.
7. Wang, Y., and Hekimi, S. (2016). Understanding Ubiquinone. Trends Cell Biol. *26*, 367–378.
8. Milewicz, D.M., Trybus, K.M., Guo, D.C., Sweeney, H.L., Regalado, E., Kamm, K., and Stull, J.T. (2017). Altered Smooth Muscle Cell Force Generation as a Driver of Thoracic Aortic Aneurysms and Dissections. Arterioscler. Thromb. Vasc. Biol. *37*, 26–34.
9. Waters, K.M., Rooper, L.M., Guajardo, A., and Halushka, M.K. (2017). Histopathologic differences partially distinguish syndromic aortic diseases. Cardiovasc. Pathol. *30*, 6–11.
10. Guo, D.C., Pannu, H., Tran-Fadulu, V., Papke, C.L., Yu, R.K., Avidan, N., Bourgeois, S., Estrera, A.L., Safi, H.J., Sparks, E., et al. (2007). Mutations in smooth muscle alpha-actin (ACTA2) lead to thoracic aortic aneurysms and dissections. Nat. Genet. *39*, 1488–1493.
11. Lino Cardenas, C.L., Kessinger, C.W., MacDonald, C., Jassar, A.S., Isselbacher, E.M., Jaffer, F.A., and Lindsay, M.E. (2018). Inhibition of the methyltranferase EZH2 improves aortic performance in experimental thoracic aortic aneurysm. JCI Insight *3*, e97493.
12. Inamoto, S., Kwartler, C.S., Lafont, A.L., Liang, Y.Y., Fadulu, V.T., Duraisamy, S., Willing, M., Estrera, A., Safi, H., Hannibal,

M.C., et al. (2010). TGFBR2 mutations alter smooth muscle cell phenotype and predispose to thoracic aortic aneurysms and dissections. Cardiovasc. Res. *88*, 520–529.
13. Li, W., Li, Q., Jiao, Y., Qin, L., Ali, R., Zhou, J., Ferruzzi, J., Kim, R.W., Geirsson, A., Dietz, H.C., et al. (2014). Tgfbr2 disruption in postnatal smooth muscle impairs aortic wall homeostasis. J. Clin. Invest. *124*, 755–767.
14. Pilop, C., Aregger, F., Gorman, R.C., Brunisholz, R., Gerrits, B., Schaffner, T., Gorman, J.H., 3rd, Matyas, G., Carrel, T., and Frey, B.M. (2009). Proteomic analysis in aortic media of patients with Marfan syndrome reveals increased activity of calpain 2 in aortic aneurysms. Circulation *120*, 983–991.
15. Jiménez-Altayó, F., Meirelles, T., Crosas-Molist, E., Sorolla, M.A., Del Blanco, D.G., López-Luque, J., Mas-Stachurska, A., Siegert, A.M., Bonorino, F., Barberà, L., et al. (2018). Redox stress in Marfan syndrome: Dissecting the role of the NADPH oxidase NOX4 in aortic aneurysm. Free Radic. Biol. Med. *118*, 44–58.
16. Chen, J., Peters, A.M., Papke, C.L., Villamizar, C., Ringuette, L.J., Cao, J.M., Wang, S., Ma, S., Gong, L., Byanova, K., et al. (2017). Loss of Smooth Muscle alpha-actin Leads to NF-κB-Dependent Increased Sensitivity to Angiontensin II in Smooth Muscle Cells and Aortic Enlargement. Circ. Res. *120*, 1903–1915.
17. Schwaerzer, G.K., Kalyanaraman, H., Casteel, D.E., Dalton, N.D., Gu, Y., Lee, S., Zhuang, S., Wahwah, N., Schilling, J.M., Patel, H.H., et al. (2019). Aortic pathology from protein kinase G activation is prevented by an antioxidant vitamin B_{12} analog. Nat. Commun. *10*, 3533.
18. van der Pluijm, I., Burger, J., van Heijningen, P.M., IJpma, A., van Vliet, N., Milanese, C., Schoonderwoerd, K., Sluiter, W., Ringuette, L.J., Dekkers, D.H.W., et al. (2018). Decreased mitochondrial respiration in aneurysmal aortas of Fibulin-4 mutant mice is linked to PGC1A regulation. Cardiovasc. Res. *114*, 1776–1793.
19. Yang, H.H., van Breemen, C., and Chung, A.W. (2010). Vasomotor dysfunction in the thoracic aorta of Marfan syndrome is associated with accumulation of oxidative stress. Vascul. Pharmacol. *52*, 37–45.
20. Oller, J., Gabandé-Rodríguez, E., Ruiz-Rodríguez, M.J., Desdín-Micó, G., Aranda, J.F., Rodrigues-Diez, R., Ballesteros-Martínez, C., Blanco, E.M., Roldan-Montero, R., Acuña, P., et al. (2021). Extracellular Tuning of Mitochondrial Respiration Leads to Aortic Aneurysm. Circulation *143*, 2091–2109.
21. Branchetti, E., Poggio, P., Sainger, R., Shang, E., Grau, J.B., Jackson, B.M., Lai, E.K., Parmacek, M.S., Gorman, R.C., Gorman, J.H., et al. (2013). Oxidative stress modulates vascular smooth muscle cell phenotype via CTGF in thoracic aortic aneurysm. Cardiovasc. Res. *100*, 316–324.
22. Vazquez Fonseca, L., Doimo, M., Calderan, C., Desbats, M.A., Acosta, M.J., Cerqua, C., Cassina, M., Ashraf, S., Hildebrandt, F., Sartori, G., et al. (2018). Mutations in COQ8B (ADCK4) found in patients with steroid-resistant nephrotic syndrome alter COQ8B function. Hum. Mutat. *39*, 406–414.
23. Lopez, L., Colan, S., Stylianou, M., Granger, S., Trachtenberg, F., Frommelt, P., Pearson, G., Camarda, J., Cnota, J., Cohen, M., et al.; Pediatric Heart Network Investigators (2017). Relationship of Echocardiographic *Z* Scores Adjusted for Body Surface Area to Age, Sex, Race, and Ethnicity: The Pediatric Heart Network Normal Echocardiogram Database. Circ. Cardiovasc. Imaging *10*, e006979.
24. Guo, D.C., Hostetler, E.M., Fan, Y., Kulmacz, R.J., Zhang, D., Nickerson, D.A., Leal, S.M., LeMaire, S.A., Regalado, E.S., Milewicz, D.M.; and GenTAC Investigators (2017). Heritable Thoracic Aortic Disease Genes in Sporadic Aortic Dissection. J. Am. Coll. Cardiol. *70*, 2728–2730.
25. Halushka, M.K., Angelini, A., Bartoloni, G., Basso, C., Batoroeva, L., Bruneval, P., Buja, L.M., Butany, J., d'Amati, G., Fallon, J.T., et al. (2016). Consensus statement on surgical pathology of the aorta from the Society for Cardiovascular Pathology and the Association For European Cardiovascular Pathology: II. Noninflammatory degenerative diseases - nomenclature and diagnostic criteria. Cardiovasc. Pathol. *25*, 247–257.
26. Sisková, Z., Mahad, D.J., Pudney, C., Campbell, G., Cadogan, M., Asuni, A., O'Connor, V., and Perry, V.H. (2010). Morphological and functional abnormalities in mitochondria associated with synaptic degeneration in prion disease. Am. J. Pathol. *177*, 1411–1421.
27. Lacro, R.V., Dietz, H.C., Sleeper, L.A., Yetman, A.T., Bradley, T.J., Colan, S.D., Pearson, G.D., Selamet Tierney, E.S., Levine, J.C., Atz, A.M., et al.; Pediatric Heart Network Investigators (2014). Atenolol versus losartan in children and young adults with Marfan's syndrome. N. Engl. J. Med. *371*, 2061–2071.
28. Widmeier, E., Yu, S., Nag, A., Chung, Y.W., Nakayama, M., Fernández-Del-Río, L., Hugo, H., Schapiro, D., Buerger, F., Choi, W.I., et al. (2020). ADCK4 Deficiency Destabilizes the Coenzyme Q Complex, Which Is Rescued by 2,4-Dihydroxybenzoic Acid Treatment. J. Am. Soc. Nephrol. *31*, 1191–1211.
29. Ejiri, J., Inoue, N., Tsukube, T., Munezane, T., Hino, Y., Kobayashi, S., Hirata, K., Kawashima, S., Imajoh-Ohmi, S., Hayashi, Y., et al. (2003). Oxidative stress in the pathogenesis of thoracic aortic aneurysm: protective role of statin and angiotensin II type 1 receptor blocker. Cardiovasc. Res. *59*, 988–996.
30. Malashicheva, A., Kostina, D., Kostina, A., Irtyuga, O., Voronkina, I., Smagina, L., Ignatieva, E., Gavriliuk, N., Uspensky, V., Moiseeva, O., et al. (2016). Phenotypic and Functional Changes of Endothelial and Smooth Muscle Cells in Thoracic Aortic Aneurysms. Int. J. Vasc. Med. *2016*, 3107879.
31. Crosas-Molist, E., Meirelles, T., López-Luque, J., Serra-Peinado, C., Selva, J., Caja, L., Gorbenko Del Blanco, D., Uriarte, J.J., Bertran, E., Mendizábal, Y., et al. (2015). Vascular smooth muscle cell phenotypic changes in patients with Marfan syndrome. Arterioscler. Thromb. Vasc. Biol. *35*, 960–972.
32. Suzuki, T., Distante, A., Zizza, A., Trimarchi, S., Villani, M., Salerno Uriarte, J.A., de Luca Tupputi Schinosa, L., Renzulli, A., Sabino, F., Nowak, R., et al.; International Registry of Acute Aortic Dissection Substudy on Biomarkers (IRAD-Bio) Investigators (2008). Preliminary experience with the smooth muscle troponin-like protein, calponin, as a novel biomarker for diagnosing acute aortic dissection. Eur. Heart J. *29*, 1439–1445.
33. Pape, L.A., Tsai, T.T., Isselbacher, E.M., Oh, J.K., O'gara, P.T., Evangelista, A., Fattori, R., Meinhardt, G., Trimarchi, S., Bossone, E., et al.; International Registry of Acute Aortic Dissection (IRAD) Investigators (2007). Aortic diameter >or = 5.5 cm is not a good predictor of type A aortic dissection: observations from the International Registry of Acute Aortic Dissection (IRAD). Circulation *116*, 1120–1127.
34. Fiorillo, C., Becatti, M., Attanasio, M., Lucarini, L., Nassi, N., Evangelisti, L., Porciani, M.C., Nassi, P., Gensini, G.F., Abbate, R., and Pepe, G. (2010). Evidence for oxidative stress in plasma of patients with Marfan syndrome. Int. J. Cardiol. *145*, 544–546.

HGGA, Volume *3*

Supplemental information

Identification of a common polymorphism in *COQ8B* acting as a modifier of thoracic aortic aneurysm severity

Benjamin J. Landis, Dongbing Lai, Dong-Chuan Guo, Joel S. Corvera, Muhammad T. Idrees, Henry W. Stadler, Christian Cuevas, Gavin U. Needler, Courtney E. Vujakovich, Dianna M. Milewicz, Robert B. Hinton, and Stephanie M. Ware

Supplemental Figures.

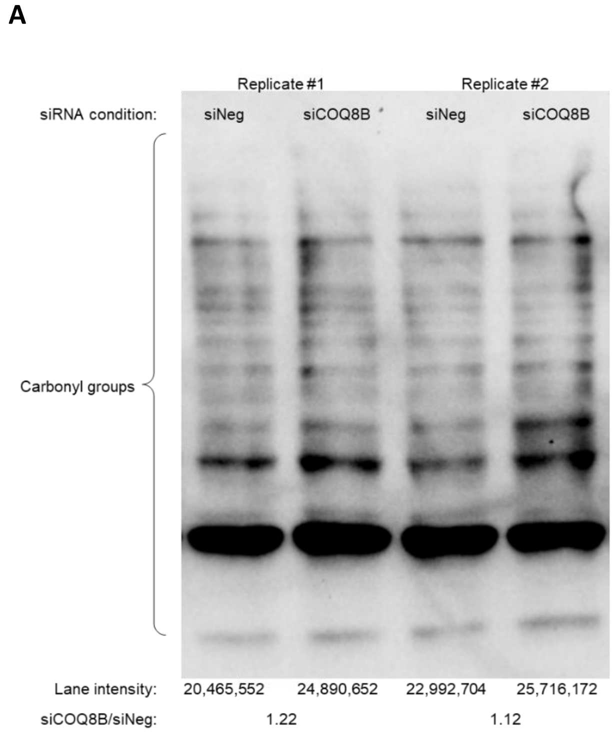

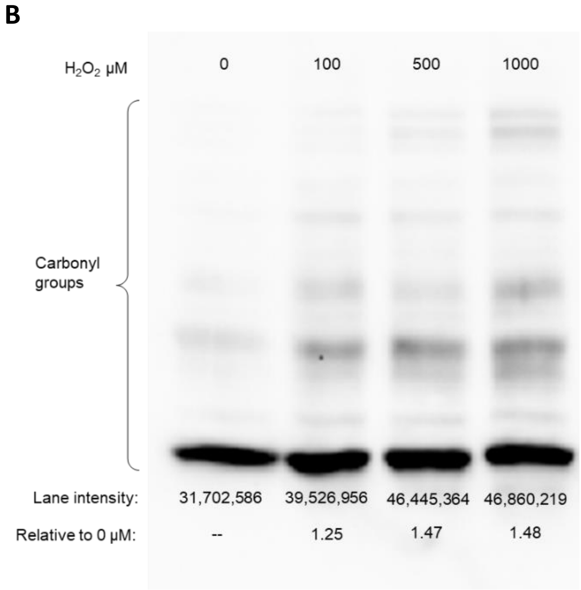

Figure S1. Replication of Oxyblot experiments and determination of assay sensitivity to oxidative stress.

(A) Protein carbonylation levels in healthy control aortic SMCs transfected with siRNA targeting COQ8B (siCOQ8B) versus negative control siRNA (siNeg). Chemiluminescent signal intensity is shown below each lane (Lane intensity). These represent biological replicates of the Oxyblot result for Control #1 SMCs that is shown in Figure 1E.

(B) Protein carbonylation levels in whole cell lysate protein samples from healthy control aortic SMCs administered different dosages of H_2O_2 (0, 100, 500, or 1000 µM) for 24 hour duration.

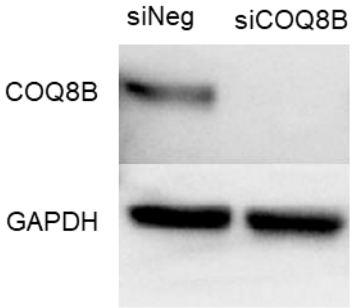

Figure S2. Western blots in whole cell lysates using a second COQ8B antibody.
Western blot for COQ8B using Atlas antibody in whole cell lysate proteins from SMCs treated with negative control siRNA versus siCOQ8B. Result confirms the specificity of LSBio antibody for COQ8B.

A

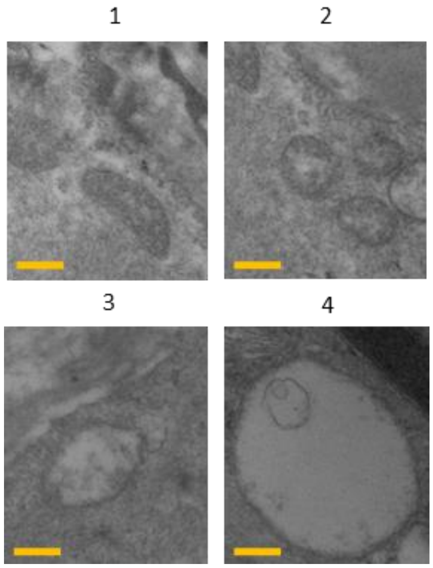

B

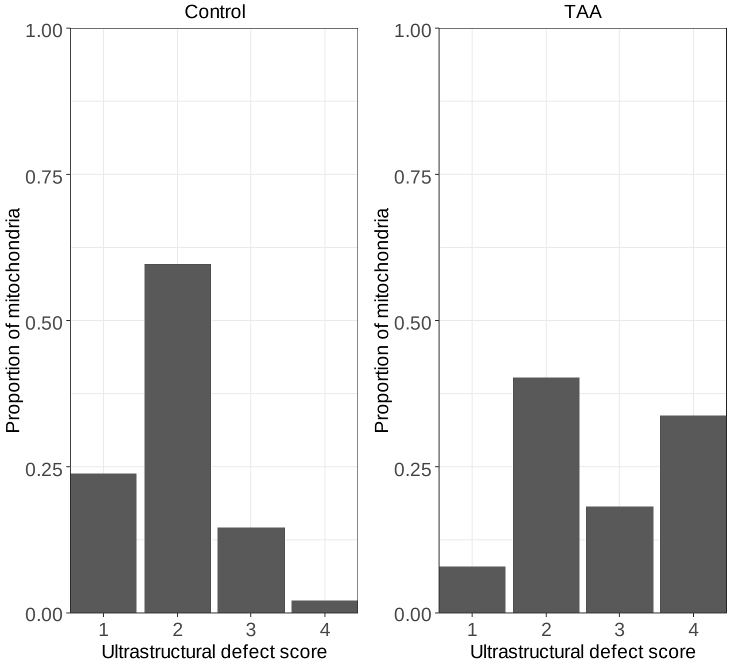

Figure S3. Scoring of ultrastructural defects of mitochondria using transmission electron microscopy of aortic tissues.

(A) Examples of mitochondria with ultrastructural defect scores of 1 (intact cristae), 2 (irregular cristae), 3 (deficient cristae), and 4 (swollen and deficient cristae).

(B) Distributions of ultrastructural defect scores in control and TAA mitochondria. Yellow bar in panel A = 250 nm.

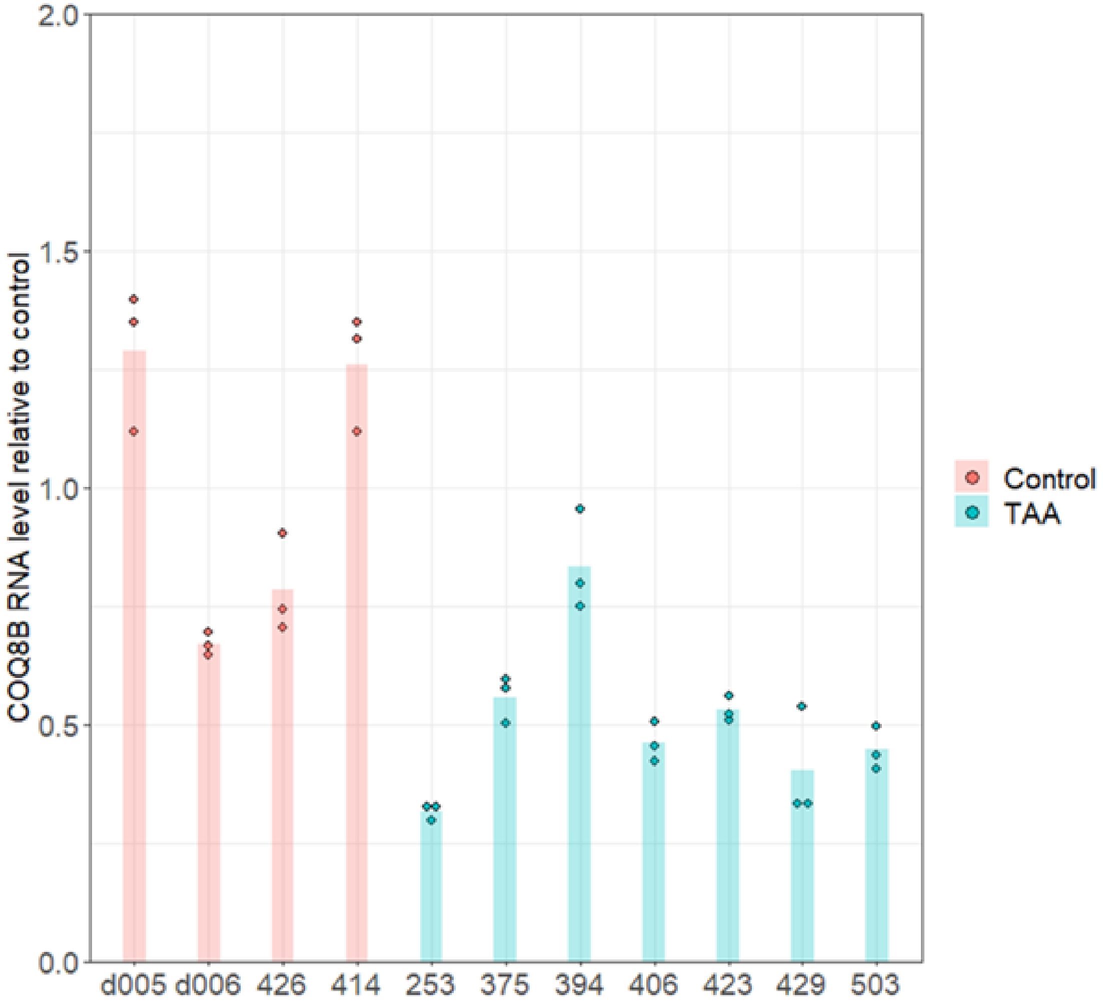

Figure S4. RNA levels of *COQ8B* between TAA (N=7 cases) and controls (N=4 individuals) in cultured proximal aortic SMCs. *COQ8B* RNA levels were adjusted for *ACTB* and are displayed relative to mean of control SMCs. Each point corresponds to an independent well of SMCs and bars indicate mean for each individual. These mean values are shown in Figure 3F.

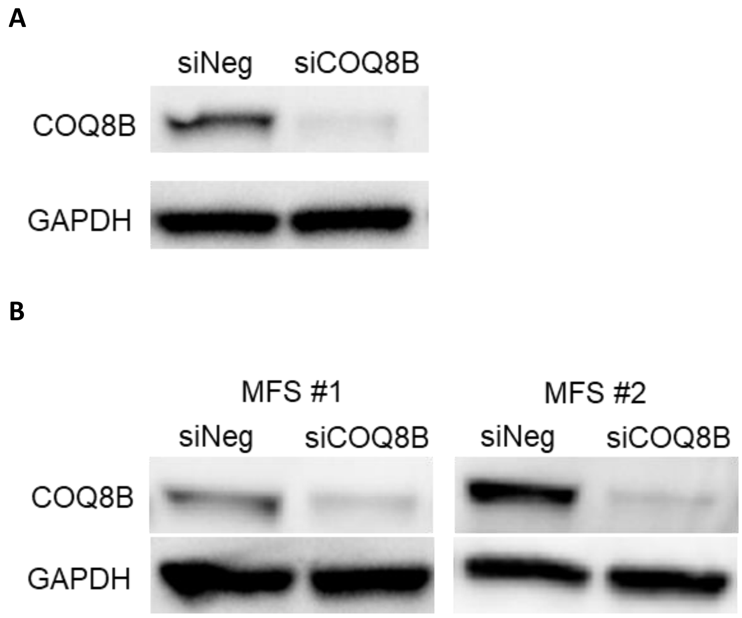

Figure S5. Western blots for COQ8B in knockdown experiments.
(A) Western blot for COQ8B in the mitochondrial protein fractions of SMCs from a participant with MFS transfected with siNeg versus siCOQ8B.
(B) Western blot for COQ8B and GAPDH in the siNeg and siCOQ8 whole cell protein samples used for Oxyblot in Figure 4A.

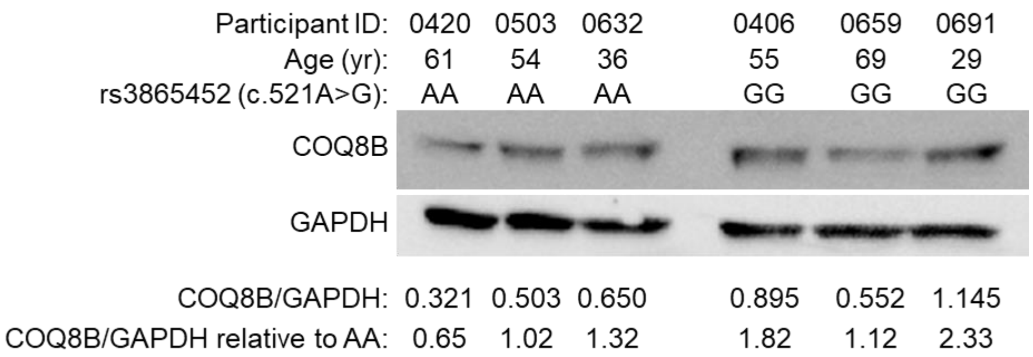

Figure S6. Western blot of aortic SMC protein lysates for 6 additional participants included in Figure 7A.

The ratios of chemiluminescent signal intensity between COQ8B and GAPDH bands are shown for each sample and also shown relative to the mean for AA samples.

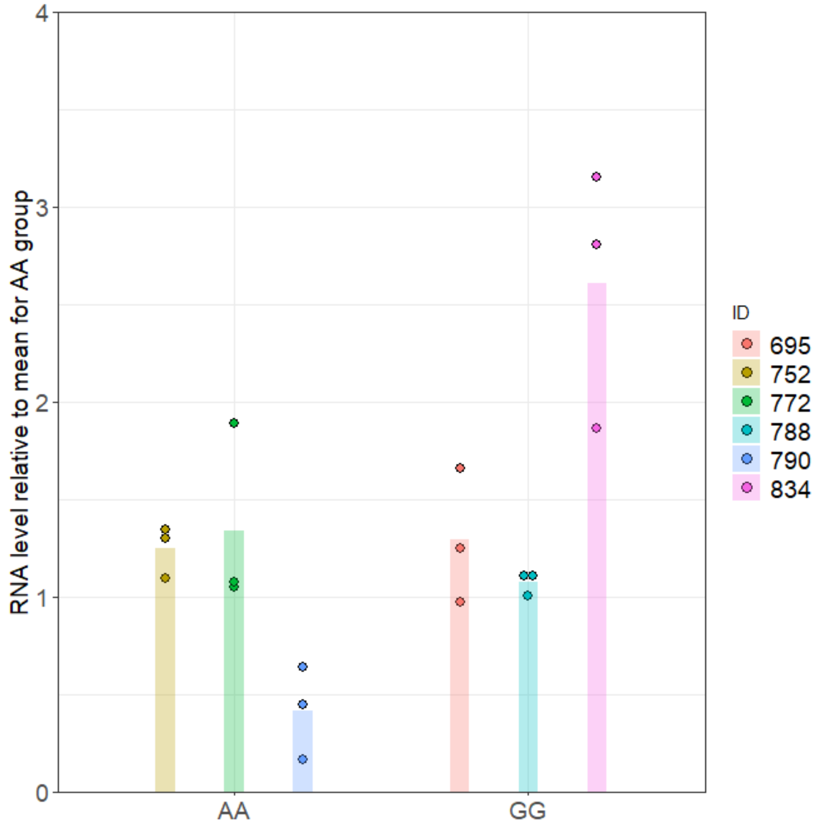

Figure S7. ***COQ8B*** **RNA levels in TAA SMCs from participants homozygous for rs3865452 (GG) or the reference allele (AA).**

RNA extractions were performed at 2nd passage in parallel with protein extractions that were used for Western blot in Figure 5. No significant difference in *COQ8B* RNA levels was observed between AA and GG groups.

Supplemental Tables.

Table S1. Primers used in qRT-PCR analysis.

Gene	Primer sequences
COQ8B	GTGTGACTACCGTCGTGAGG<br>GGAAGAAGGGGTCATTTGC
ACTA2	CTGTTCCAGCCATCCTTCAT<br>TCATGATGCTGTTGTAGGTGGT
CNN1	GCTGTCAGCCGAGGTTAAGA<br>CCCTCGATCCACTCTCTCAG
MYOCD	CAAGTTCAGCAATTTCAGAGGTAA<br>TGACTCCGGGTCATTTGC

Table S2. Clinical and demographic data of participants whose ascending aortic tissues were studied with transmission electron microscopy.

Characteristic	Control (N=3)	TAA (N=7)
Age (years), mean ± standard deviation	36 ± 25	58 ± 10
Sex, N		
Male	3	6
Female	0	1
Race, N		
White	2	6
Black	0	1
Data not available	1	0
Family history of TAA, N	0	3
Thoracic aortic dissection, N	0	1
Aortic valve morphology, N		
Tricuspid	3	5
Bicuspid or unicuspid	0	2
Hypertension, N	0	3
Dyslipidemia, N	1	2
Type 2 diabetes, N	1	0
Coronary artery disease, N	1	1

TAA: thoracic aortic aneurysm

Table S3. Clinical and demographic data of participants whose samples were used to compare *COQ8B* RNA levels in primary culture aortic SMCs between TAA and controls.

Characteristic	Control (N=4)	TAA (N=7)
Age, years (mean ± standard deviation)	26 ± 10	61 ± 13
Sex, N		
Male	4	3
Female	0	4
Race, N		
White	2	6
Black	0	1
Data not available	2	0
Family history of TAA, N	0	1
Thoracic aortic dissection, N	0	0
Aortic valve morphology, N		
Tricuspid	3	4
Bicuspid or unicuspid	1	3
Hypertension, N	1	7
Dyslipidemia, N	0	3

TAA: thoracic aortic aneurysm.

Table S4. Cardiovascular characteristics of patients in longitudinal TAA cohort used for mixed model analysis.

Characteristic	MFS or LDS (N=23)	Nonsyndromic (N=25)
Initial aortic root Z-score, mean ± standard deviation	3.8 ± 1.8	3.5 ± 1.7
Initial ascending aorta Z-score, mean ± standard deviation	0.5 ± 1.4	2.8 ± 2.0
Age at initial echo (years), mean ± standard deviation	10.1 ± 5.9	9.9 ± 4.1
Duration of follow up (years), mean ± standard deviation	5.6 ± 4.9	5.1 ± 3.2
Number of echocardiograms, mean ± standard deviation	7.4 ± 4.6	6.4 ± 3.4
Bicuspid aortic valve, N	3	11
Aortic replacement surgery, N	2	1
Aortic dissection, N	1	0
